# Single‐Dose TACE‐Like Injection of Sustained‐Release Panobinostat/IL‐12 Polymeric Microparticles is Effective in Preclinical Liver Cancer Models

**DOI:** 10.1002/advs.77223

**Published:** 2026-08-24

**Authors:** Hongzhe Yu, Ling Li, Sachin S. Surwase, Xin Ming M. Zhou, Hongtao Qian, Joel C. Sunshine, Florin M. Selaru, Jordan J. Green

**Affiliations:** ^1^ Department of Biomedical Engineering Translational Tissue Engineering Center Johns Hopkins Translational ImmunoEngineering Center and the Institute for Nanobiotechnology Johns Hopkins University Baltimore Maryland USA; ^2^ Division of Gastroenterology and Hepatology Department of Medicine Johns Hopkins University Baltimore Maryland USA; ^3^ Department of Dermatology Johns Hopkins University Baltimore Maryland USA; ^4^ Department of Oncology Bloomberg‐Kimmel Institute for Cancer Immunotherapy and Sidney Kimmel Comprehensive Cancer Center Baltimore Maryland USA; ^5^ Departments of Materials Science and Engineering Chemical and Biomolecular Engineering, Ophthalmology, and Neurosurgery Johns Hopkins University Baltimore Maryland USA

**Keywords:** HCC, IL12, panobinostat, PLGA/PBAE microparticle, TACE

## Abstract

**Background & Aims**: Primary liver cancer is the third leading cause of cancer death worldwide, and about 70% of hepatocellular carcinomas (HCCs) do not respond to systemic immunotherapies. Transarterial chemoembolization (TACE) remains standard treatment for intermediate‐stage HCC, but long‐term survival is poor. We aimed to develop a polymeric TACE‐like platform to sensitize liver cancer to checkpoint inhibitors by enabling sustained co‐delivery of panobinostat and IL‐12.

**Methods**: Patient‐derived liver cancer organoids were used to screen drugs and identify panobinostat as a cytotoxic agent. We then formulated sustained‐release microparticles from anionic poly(lactic‐co‐glycolic acid) (PLGA) and cationic poly(beta‐amino ester) (PBAE) for combined delivery of panobinostat and IL‐12, and tested the system in three liver cancer models.

**Results**: The microparticles achieved tumor‐specific retention and sustained release for two weeks following a single intratumoral or intrahepatic arterial injection. This led to tumor inhibition and prolonged survival in orthotopic liver cancer models. Mechanistically, treatment promoted tertiary lymphoid structure formation, increased infiltration of immune cells, and elevated pro‐inflammatory cytokines, triggering innate and adaptive antitumor immune responses.

**Conclusions**: A PLGA/PBAE sustained‐release microparticle platform was successfully developed for TACE‐like co‐delivery of panobinostat and IL‐12. This combined chemoimmunotherapy strategy shows promise for overcoming immune suppression and improving liver cancer treatment.

## Graphical abstract

A novel PLGA/PBAE polymer microparticle platform revolutionizes intermediate‐stage liver cancer treatment by co‐delivering panobinostat and IL‐12 via a single TACE‐like injection. This sustained‐release system overcomes tumor immunosuppression, triggers robust innate and adaptive immune responses, and establishes tertiary lymphoid structures. Ultimately, this approach inhibits tumors and extends survival, offering a promising strategy to sensitize liver cancers to immunotherapy.

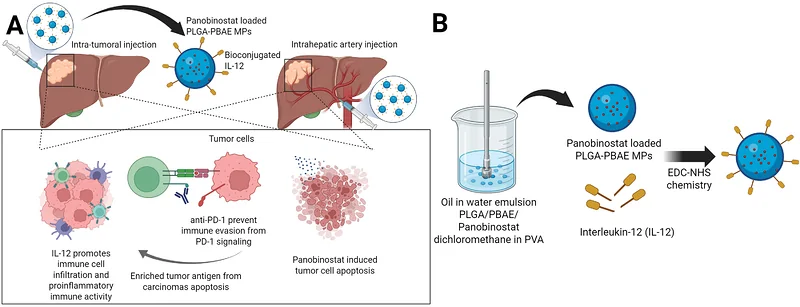

## 1 Introduction

Primary liver cancer is the sixth most common cancer and the third leading cause (7.8%) of cancer mortality worldwide [1]. According to the World Health Organization (WHO), liver cancer will contribute to more than one million deaths in 2030 [2]. HCC prognosis is poor, with a 5‐year survival rate of 18% [2]. Immune checkpoint inhibitors (ICIs) have improved outcomes in advanced HCC [3, 4, 5, 6]. For early‐intermediate stage HCC and selected advanced unresectable HCC with preserved liver function, transarterial chemoembolization (TACE) has been the standard for > 20 years and is utilized in 50–60% HCC; yet most progress within 1 year [7], and the long‐term survival outcomes are unsatisfactory [8, 9]. With the rise of immunotherapy for HCC, two global phase 3 clinical trials of EMERALD‐1 (TACE + durvalumab ± bevacizumab) and LEAP‐012 (TACE + lenvatinib + pembrolizumab [10, 11], showed longer progression‐free survival compared with TACE alone, indicating that TACE plus systemic ICI and target therapy holds promise for intermediate‐stage HCC. However, nearly all patients in the trials had some adverse events with ∼28% of patients withdrawing [10, 11]. In addition, due to the immunosuppressive tumor microenvironment (TME), tumors with some degree of inflammation constitute only ∼30% HCC. More than 70% “cold” HCC are much less likely to benefit from current systemic ICIs [12]. A strategy to turn “cold” tumors into “hot” tumors holds the promise of leveraging the benefit of ICI in a higher percentage of HCC patients. Panobinostat, a pan‐histone deacetylase inhibitor (HDACi), was reported to exhibit cell cytotoxicity in several cancers [13, 14]. However, in prior clinical trials, panobinostat did not show high efficacy in HCC [15], likely due to (1) poor tumor site drug delivery with oral administration, as well as (2) the presence of cold TME. This highlights the need for a targeted, high‐dose sustained‐release delivery approach. In addition to cell cytotoxicity, panobinostat has also been reported to upregulate MHC I/II, differentiation antigens, costimulatory factors [16, 17, 18], and PD‐L1 [19, 20]. Therefore, we hypothesized that we could achieve even higher efficacy by leveraging the increased tumor sensitivity to immunotherapy induced by panobinostat. These data support the rationale for combining HDAC inhibitors with immunotherapy to achieve additive or synergistic antitumor effects.

Interleukin‐12 (IL‐12), produced by activated antigen‐presenting cells, is a potent, pro‐inflammatory cytokine. IL‐12 plays a critical role in innate and adaptive immunity [21]. IL‐12 can promote the proliferation and cytotoxic ability of natural killer (NK) cells, NKT and T cells, and drive the polarization of Th1 effector cells to enhance IFN‐γ secretion, leading to upregulated MHC class I and II expression on tumor cells and improved immune recognition and tumor lysis [21, 22]. In addition, IL‐12 can inhibit or reprogram immunosuppressive cells, including Treg, tumor‐associated macrophages, and myeloid‐derived suppressor cells [23, 24, 25]. Furthermore, IL‐12 has direct effects on dendritic cells to improve antigen presentation to increase IL‐12 production [26]. Last, IL‐12 has shown robust antitumor activity in preclinical studies [22, 27]. However, clinical trials have been disappointing, likely due to both insufficient dosage of IL‐12 in the local TME and potentially severe toxicity of systemically administered IL‐12 in patients [28, 29, 30].

Polymeric microparticles (MPs) have shown capabilities of drug encapsulation, sustained release, and extended in vivo retention previously [31]. Moreover, immunomodulatory protein‐functionalized polymeric micro‐ and nanoparticles, such as artificial antigen‐presenting cells (aAPCs) [32] and cytokine delivery particles [33] have been investigated and shown to modulate immune cells. Biodegradable poly (lactic‐co‐glycolic acid) (PLGA) has also been investigated as a drug delivery biomaterial for various small molecule drugs as a result of its biocompatibility, controlled degradation, and extended in vivo retention [34]. PLGA micro‐ and nanoparticles have shown utility as aAPC core materials for immune cell interaction and presenting stimulation signals to T cells [32, 35]. Cationic poly (beta‐amino ester) (PBAE) was incorporated with PLGA as aAPCs and polymer blends of anionic PLGA and cationic PBAE showed increased protein conjugation efficiency and improved functionality compared to PLGA‐based aAPCs [36]. Polymeric particles of PLGA‐PBAE blends are a potential co‐delivery platform of small molecules and protein for a single‐dose loco‐regional therapy. However, their therapeutic efficacy for combination TACE therapy, their potential extended in vivo retention, and their sustained release to various orthotopic liver cancers have not been investigated.

Here, we report MPs composed of biodegradable polymer blends of anionic PLGA and cationic PBAE that enable co‐delivery of Panobinostat and IL‐12 to orthotopic liver cancer locally via locoregional intratumor and intrahepatic artery injection. The PLGA‐PBAE formulation can localize Panobinostat and IL‐12 to the TME and release both agents in a sustained fashion for at least 15 days without any systemic toxicity. One dose of the PLGA‐PBAE MPs plus anti‐PD‐1 therapy can inhibit tumor growth and elicit significant overall survival benefit in both mouse and rat orthotopic HCC and CCA models. Using CODEX (PhenoCycler) multiplexed imaging, flow cytometry, cytokine detection, and transcriptome analysis, we demonstrate that local immunologic activation is achieved for both innate and adaptive immunity that promotes the effective elimination of orthotopic liver cancer.

## 2 Materials and Methods

### 2.1 Study Design

This study was designed to evaluate a novel combination therapy based on a polymeric particle‐based single‐dose loco‐regional treatment in liver cancers. Preclinical animal models of orthotopic HCC and CCA were used to investigate in vivo retention and biodistribution of biodegradable polymeric MPs, therapeutic efficacy, impact on innate and adaptive antitumor immune responses, and overall survival. The indicated sample size (*n*) represents biological replicates unless otherwise noted in the figure legends. All experiments were repeated independently two or three times. All samples that met proper experimental conditions were included in the analysis. All in vivo animal experiments were approved by the Johns Hopkins University Animal Care and Use Committee (ACUC). Animals were randomized based on tumor burden measured using calipers to achieve similar tumor size across experimental groups. Tumors that did not reach the size of 2 mm × 2 mm before the particle injections were excluded from experiments based on pre‐established criteria. For survival studies, mice that developed ascites were euthanized according to ACUC guidelines. For in vitro experiments, sample allocation was performed randomly.

### 2.2 HCC and CCA Patients and Samples

Fresh human HCC and CCA specimens for organoid studies were collected at the surgery department of the Johns Hopkins Hospital with informed consent approved by the Institutional Review Board of the Johns Hopkins University. Human liver cancer organoid culture, drug screening, and PDX establishment are described before [37, 38].

### 2.3 Hydrophobic Poly(Beta‐Amino‐Ester) (PBAE) Synthesis

Hydrophobic poly(beta‐amino‐ester) (PBAE) was synthesized via Michael addition of diacrylate monomer with amine‐containing monomers as previously described [36]. In brief, 4,4’‐trimethylenedipiperidine and 1,4‐butanediol diacrylate at stoichiometric ratios of 1.2:1 acrylate: amine monomers were dissolved in anhydrous dimethylformamide (DMF), and the reaction proceeded for 24 h at 85°C under stirring. The obtained acrylate‐terminated polymer was dissolved together with an end‐capping monomer 1‐(3‐aminopropyl)‐4‐methylpiperazine at 0.5 M endcap monomer and 200 mg/mL of base polymer in tetrahydrofuran (THF) and the reaction proceeded for 1 h at room temperature to form the final polymer. The obtained polymers were purified by precipitation in hexane and two washes. The polymers were dried under vacuum for 48 h and stored as aliquots at −20°C with desiccant.

### 2.4 Polymeric MP Synthesis

Polymeric nanoparticles were synthesized using single emulsion techniques as previously described with modifications [32, 36]. Briefly, poly (lactic‐co‐glycolic acid) acid terminated (38–54 kDa, 50:50 L:G ratio) (PLGA, Sigma Aldrich; St. Louis, MO) or a blend of PLGA and PBAE with a molecular weight of 500–30 kDa at 75:25 wt/wt ratio was dissolved at 20 mg/mL in 5 mL dichloromethane (DCM) and homogenized by a T‐25 digital ULTRA‐TURRAX IKA tissue homogenizer (IKA Works; Wilmington, NC) in 50 mL of 1% polyvinyl alcohol (PVA) solution to generate emulsification. The resulting emulsification was poured into 100 mL of a 0.5% PVA solution on a magnetic stir plate at 500 rpm overnight to allow the particles to harden. The particles were collected and washed three times with deionized water at 3,000 g for 5 min, then frozen and lyophilized. For panobinostat‐loaded MPs, panobinostat was dissolved in dimethylsulfoxide (DMSO) at 100 mg/mL and then added to polymer solutions in DCM before emulsification at a 20% wt/wt ratio. For in vivo biodistribution studies, particles were synthesized encapsulating a carboxylic acid‐terminated Cyanine7.5 dye (Lumiprobe; Hunt Valley, MD) at a 2% wt/wt ratio.

### 2.5 Interleukin 12 (IL‐12) Conjugated Particles Synthesis

IL‐12 was conjugated to polymeric MPs via carbodiimide cross‐linking chemistry with a previously developed protocol [32, 36]. Briefly, particles were activated in 0.1 M MES buffer at pH 6.0 in the presence of 1‐ethyl‐3‐(‐3‐dimethylaminopropyl) carbodiimide (EDC; Sigma‐Aldrich; St. Louis, MO) and N‐hydroxysulfosuccinimide (NHS; Sigma‐Aldrich; St. Louis, MO) for 30 min at room temperature, then incubated overnight at 4°C with recombinant mouse IL‐12 (p70) (Biolegend; San Diego, CA). The resulting particles were then washed with PBS at 3,000 g for 5 min, three times, to remove unconjugated IL‐12 before later use.

### 2.6 Characterization of IL‐12 Conjugated, Panobinostat‐Loaded MPs

Panobibostat‐loaded particles were characterized by transmission electron microscopy (TEM) using a Hitachi 7600 TEM (Hitachi High‐Tech; Tokyo, Japan). The particles were also characterized by dynamic light scattering (DLS) using a Malvern Panalytical ZetaSizer Pro (Malvern Panalytical Inc.; Westborough, MA) for the measurement of hydrodynamic diameter and zeta potential. The particles were suspended at a concentration of 1 mg/mL in 0.5X PBS in a Universal ‘Dip’ Cell Kit (Malvern Panalytical Inc.; Westborough, MA) using recommended machine settings. To evaluate the loading efficiency of panobinostat, 2 mg panobinostat‐loaded PLGA or PLGA/PBAE MPs were dissolved in DMSO, and the absorbance at 290 nm was measured on a Varioskan Multimode Microplate Reader (Thermo Scientific; Waltham, MA). The concentration of panobinostat loaded in the particles was calculated from standard absorbance curves of known concentrations of panobinostat. For the characterization of the release profile of panobinostat, an established method was adapted [39]. Briefly, particles were incubated in PBS at 37°C on an inverter. At various time points, particles were centrifuged, and supernatants were collected and diluted 1:1 in DMSO, and the absorbance was measured at 290 nm. The conjugation and release of IL‐12 was evaluated by a BCA assay. In brief, IL‐12 conjugated particles were incubated in PBS at 37°C on an inverter, and the supernatants were collected after centrifugations at various time points. The protein amounts of supernatants and freshly IL‐12 conjugated particles were measured using BCA assays (ThermoFisher; Agawam, MA) following the manufacturer's protocols.

### 2.7 Animal Experiments

All animal experiments were approved by the Animal Care and Use Committee (ACUC) of Johns Hopkins University. For all surgical procedures, animals were anesthetized with 1%–3% isoflurane in 100% oxygen until a sufficient plane of anesthesia was reached. During surgical procedures, animals were placed onto a 37°C heated surface to ensure optimum thermoregulation and minimize post‐op recovery time. The hair was removed from the surgical site (abdomen) using clippers and/or depilatory cream and prepared using a wash of povidone‐iodine soap, then rinsed with 70% ethanol following the standard protocol. After surgery, the peritoneum was closed using simple continuous suturing of the muscle layer with absorbable sutures, and the skin layer was closed using simple interrupted suturing with non‐absorbable sutures. Warm, sterile isotonic fluids at 3%–5% of the body weight were injected subcutaneously before and at the end of surgery. Meloxicam (2 mg/kg) was delivered subcutaneously before surgery and again at 24 and 48 h after surgery.

### 2.8 Evaluation of In Vivo Pharmacokinetics

For mouse studies, PLGA/PBAE particles (*n* = 4) encapsulating a near‐IR hydrophobic dye Cy7.5 were suspended at 200 mg/mL in sterile PBS, and 25 µL of the particle suspension was injected intratumorally into orthotopic liver tumors of C57BL/6J mice (The Jackson Laboratory; Bar Harbor, ME). For rat studies, the Cy7.5‐labelled PLGA/PBAE particles were injected through the gastroduodenal artery toward the common hepatic artery of orthotopic N1‐S1 tumor‐bearing Sprague‐Dawley rats (SD rats) (Charles River Laboratories; Wilmington, MA). The fluorescence intensity was measured by an IVIS Spectrum optical imaging scanner (Spectral Instruments Imaging; Tucson, AZ) at different time points up to 15 days. On 15 days post administration, the animals were sacrificed and the liver, spleen, heart, and lungs were dissected out and imaged on the IVIS scanner.

### 2.9 Evaluation of In Vivo Therapeutic Efficacy in Mouse Models

For in vivo evaluation of antitumor efficacies of PPP‐IL12, orthotopic liver tumors were implanted to C57BL/6J mice by an established method [40, 41]. Hepa1‐6 (for HCC) and SB1 (for CCA) cells were injected subcutaneously into the right flanks of C57BL/6J mice. After 2–3 weeks, the tumor masses were harvested and dissected into small fragments. The HCC fragments were then implanted into the liver lobes of healthy C57BL/6J mice. The particle treatments were administered 2 weeks later intratumorally and consisted of either PBS, PPP, IL‐12 conjugated empty MPs (PP‐IL12), or PPP‐IL12 (*n* = 4–7 mice/group, 5 mg particles/mouse, which include 1 mg panobinostat and 10 µg IL‐12 in MP specifically). Initially, tumor‐burden mice are randomly grouped for treatment; each group contains 7 mice. However, while we performed the orthotopic intertumoral drug injection, drugs leaked out from some treated mice, these mice were then removed out from the study. In addition, some mice died after the treatment surgery. Which end up with *n* = 4–7. Anti‐PD‐1was administered intraperitoneally twice a week at 200 µg/mouse. The animals were sacrificed, and tumors were measured by calipers 14 days post particle administration. For survival studies, the animals were monitored every day from day 15 following the particle injection for symptoms of ascites and/or health deterioration as the benchmark for euthanasia. Animals that survived past 90 days were considered long‐term survivors and were found to be healthy 180 days following the start of the experiment. The organs of long‐term survivors were harvested and prepared for H&E staining for pathological analysis.

### 2.10 Orthoptic HCC Rat Model Establishment and Intrahepatic Artery Drug Delivery

Orthotopic HCC was implanted into Sprague‐Dawley rats (SD rats) by an established method [41]. Briefly, a laparotomy was performed, and 5×10^6^ N1‐S1 HCC cells in a 1:1 solution of HBSS and Matrigel Matrix (Corning, NY) were injected under the liver capsule. Successful inoculation of HCC cancer was validated by a white protrusion at the point of injection. Gentle compression was applied for at least 15 s using a sterile cotton‐tipped applicator to prevent cancer cell leakage or bleeding. To prevent the spontaneous tumor regression in SD rats, cyclosporine A (20 mg/kg/day) was subcutaneously injected from one day before and 4 days after the tumor cell implantation for a total of 6 days [42]. The treatments of PBS or PPP‐IL12 were administered 10 days later via intrahepatic artery injection (*n* = 5–8 rats/group, 9 mg MPs per rat, the MPs contain 1.8 mg panobinostat and 10 µg IL12).

For intrahepatic artery injection, the procedure was modified from a microsurgical hepatic artery injection [38, 41, 43]. Animals were prepared for sterile technique surgery as described above, a midline laparotomy was performed, and the lateral left lobe of the liver was externalized on a sterile piece of gauze soaked with saline and retracted. The mesenteric connective tissues were dissected, and the common bile duct was retracted using a silk suture to allow visualization of the common hepatic artery, proper hepatic artery, and gastroduodenal artery (GDA). The GDA was freed from surrounding tissue and ligated at the distal end. A few drops of 2% lidocaine were applied to the GDA to promote vasodilation. The common hepatic artery was clamped using a bulldog clamp, occluding blood flow. A 27‐gauge needle was inserted into the gastroduodenal artery, and 500 µL of PBS or PPP‐IL12 was slowly injected at a rate of 100 µL per minute. Successful injection was confirmed by visualizing the displacement of blood in the proper hepatic artery. Following the injection, the needle was slowly withdrawn from the vessel. A suture was used to ligate the GDA proximal to the needle insertion site, and the clamp was removed from the common hepatic artery to restore blood flow through the proper hepatic artery. The liver lobe was replaced in the abdominal cavity, and the incision was closed as described above. Anti‐PD‐1 was administered intraperitoneally twice a week at 1.6 mg/rat. The animals were sacrificed, and the tumor size was measured 14 days post‐MPs administration.

### 2.11 Evaluation of In Vivo Immune Responses of Combination Therapy

In order to characterize the immune responses following the combination therapy, mice were inoculated with Hepa1‐6 tumors following the procedure above, and particles and anti‐PD‐1 were administered as described above. Mice were euthanized 7 days following particle injection, and tumors and spleens were excised for ex vivo analysis. Tumor samples were lysed and analyzed using the BioLegend LEGENDplex Mouse Inflammation Panel (13‐plex) kit (BioLegend; San Diego, CA). Para‐cancerous tissue was dissociated physically, and red blood cells were lysed using ACK buffer (Quality Biological; Gaithersburg, MD), and samples were filtered and single cells stained for analysis via flow cytometry using the antibodies in Supplementary Table S2. Flow cytometry was performed on an Attune NxT Flow Cytometer (ThermoFisher Scientific; Waltham, MA) and SH800S cell sorter (Sony Biotechnology, San Jose, CA), and FlowJo software (FlowJo LLC; Ashland, OR) was used for data analysis.

### 2.12 CODEX (PhenoCycler) Multiplexed Imaging and Single‐Cell Analysis

Orthotopic HCC was harvested at the end point of treatment, fixed in 10% neutral buffered formalin, and embedded/ sectioned by the Johns Hopkins Oncology Tissue Services core. H&E staining was performed by the Johns Hopkins Oncology Tissue Services core using standard protocols. To image HCC samples, CODEX multiplexed images were used according to an established protocol [44, 45]. It is a technique that uses iterative imaging and DNA barcoded antibody labels to enable imaging of multiple antibodies simultaneously. Briefly, murine FFPE samples were sectioned onto poly‐l‐lysine–treated microscope slides and baked at 60°C overnight inside an incubator, deparaffinized with xylenes, rehydrated with a series of alcohol solutions from 100% to 30% concentration, and washed twice with Milli‐Q ultrapure water. Samples were then incubated in an Instant Pot pressure cooker at high pressure with AR9 buffer (Akoya Biosciences; Marlborough, MA) for antigen retrieval. Tissues were incubated in a cocktail solution of all custom‐conjugated antibodies (Supplementary Table S1) overnight. The next day, tissues were incubated in a post‐staining fixing solution, followed by ice‐cold methanol and fixative reagent. Fixed tissue slides were then assembled into flow cells for imaging on the PhenoCycler‐Fusion 2.0 device (Akoya Biosciences; Marlborough, MA).

To obtain quantitative single‐cell data, cell segmentation was performed in the digital pathology analysis software QuPath. Proteomics data was processed and analyzed in R and R Studio. Clustering and phenotyping were performed in the R package FlowSOM v2.13.9, with scripts modified from https://github.com/xzhou125/JOVE_QuPath_Spatial_Analysis [45].

### 2.13 RT‐PCR Array and Pathway Analysis

Total RNA was extracted from bulk HCC tumor tissue using the Trizol reagent (Qiagen) and purified with RNeasy kit (Qiagen). cDNA was obtained using RevertAid RT Reverse Transcription Kit (ThermoFisher). Mouse Cancer Inflammation & Immunity Crosstalk RT^2^ Profiler PCR Array (Qiagen) was applied, and Real‐time quantitative polymerase chain reaction (qPCR) was performed in triplicate using SYBR Green PCR Master Mix (Applied Biosystems) on the QuantStudio 3 Real‐time PCR System (Applied Biosciences). The comparative CT method (2^−∆∆CT^) was used to determine fold differences between the target gene and the reference gene GAPDH. Mouse Cancer Inflammation & Immunity Crosstalk RT^2^ Profiler PCR Array primer sequences are listed in Supplementary Table S3. Pathway analysis was performed on the PubMed Database for Annotation, Visualization, and Integrated Discovery (DAVID) following an established method [46].

### 2.14 Statistics

All experiments were performed with *n* = 3–7 replicates unless otherwise stated. Bar graphs indicate mean ± standard error of the mean; not significant (n.s.) indicates *p* > 0.05, * indicates *p* ≤ 0.05, ** indicates *p* ≤ 0.01, *** indicates *p* ≤ 0.001, and **** indicates *p* ≤ 0.0001. All statistics were performed using statistical analysis software modules in GraphPad Prism 8 (GraphPad, La Jolla, CA). For drug loading, protein conjugation, particle size, and zeta potential measurements, a Student's *t*‐test was used to assess the difference between PLGA and PLGA‐PBAE particles. For mouse HCC tumor size measurements, a Brown‐Forsythe and Welch ANOVA and a two‐stage linear step‐up procedure of Benjamini, Krieger, and Yekutieli multiple comparisons test were used for the analysis. For mouse CCA tumor size measurements, multiple t‐tests with a two‐stage linear step‐up procedure of Benjamini, Krieger, and Yekutieli were used to assess the treatment effect. For rat HCC tumor size measurements, a two‐tailed Mann‐Whitney test was used to assess the treatment effect. For mouse and rat particle biodistribution studies, an ordinary one‐way ANOVA and Dunnett's multiple comparisons test were applied. For transcription levels of cytokines in tumor lysis measures, a two‐tailed unpaired *t*‐test was performed to compare the untreated control and PPP‐IL12 + anti‐PD‐1 groups. For immune cell profiling, an ordinary one‐way ANOVA and Dunnett's multiple comparisons test were used for the analysis. For cytokine level measurements, Brown‐Forsythe and Welch ANOVA and Dunnett's multiple comparisons test were used for the analysis. In all cases, differences were considered significant if the *p*‐value of the test was less than 0.05.

## 3 Results

### 3.1 Panobinostat is Effective in Human HCC PDOs and PDO‐Derived PDX Models

**FIGURE 1:**
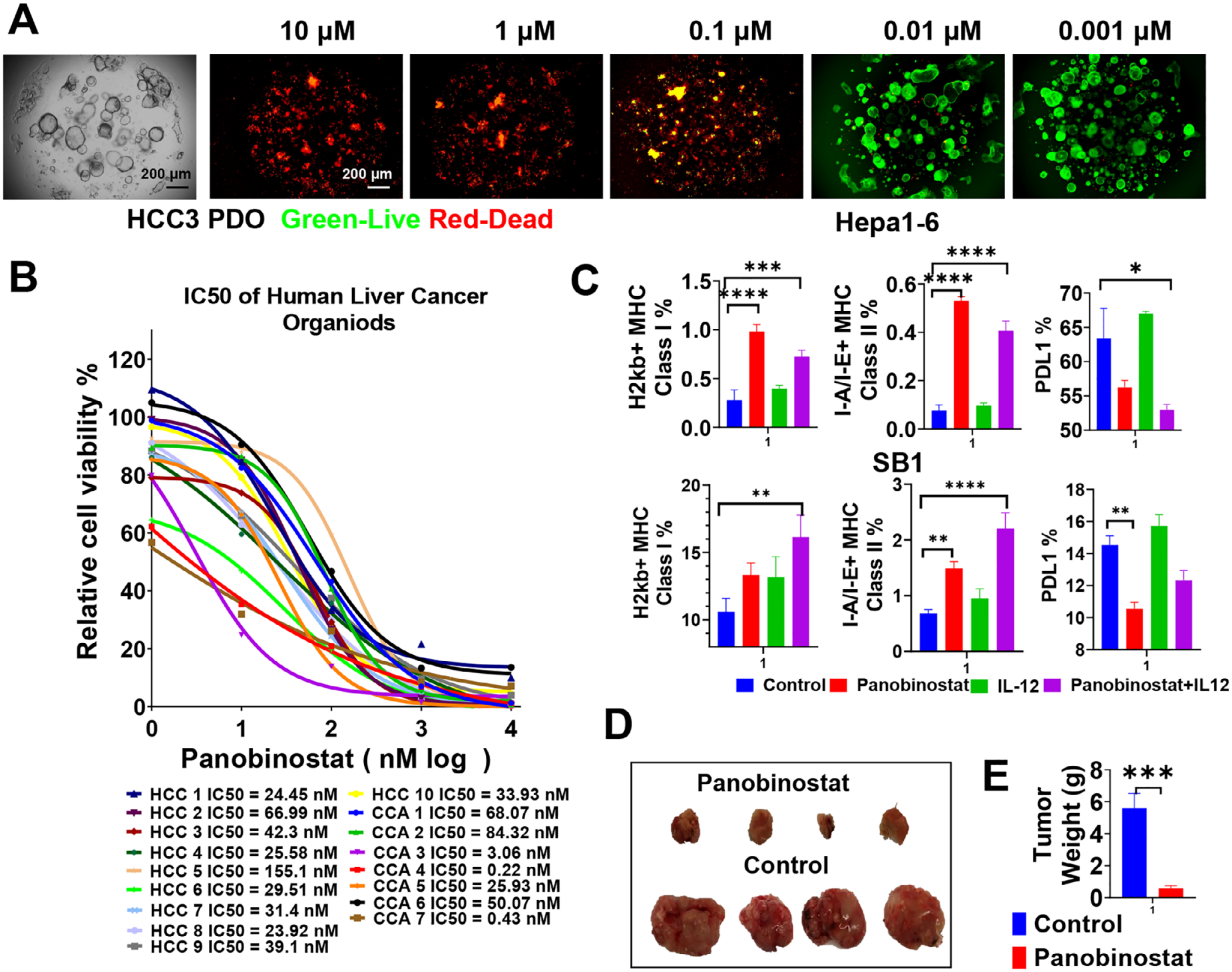
Panobinostat is effective against human HCC PDOs in vitro and PDO‐derived PDX in vivo. (A) Live/Dead staining of HCC PDOs treated with panobinostat. (B) IC50 of 17 liver cancer PDO lines treated with panobinostat. (C) Flow cytometry analysis of class I, II MHC, and PD‐L1 in Hepa1‐6 and SB cell lines treated with panobinostat, IL‐12, as well as Panobinostat + IL‐12. (*n*=3) The results indicated that panobinostat and panobinostat+IL12 treatment upregulated MHC I/II, while panobinostat+IL12 treatment downregulated PD‐L1 in Hepa1‐6 cells. While panobinostat+IL12 upregulated both MHC I/II, panobinostat alone inhibited PD‐L1 in SB1 cells. Error bars, mean ± SEM. P values were calculated using one‐way ANOVA with Tukey's posttests. *p < 0.05, **p < 0.01, ***p < 0.001, ****p < 0.0001. (D) Gross image of HCC PDO‐derived PDX treated with panobinostat vs control. (E) Measured tumor weight. (*n*=4). Error bars, mean ± SD. *P* values were analyzed using a student's *t*‐test. *p < 0.05, ***p < 0.001.

Previously, we generated 40 liver cancer patient‐derived organoids (PDO) lines and screened for effective HCC therapies with an NCI cancer drug library; panobinostat was a top hit at 10 µM [37, 38]. We further found that most of the IC50s were <100 nM in 17 PDOs (Figure 1A,B). By repeated i.t. dosing of panobinostat in PDO‐derived PDX models, we found significant tumor inhibition (Figure 1D,E). To characterize the effects of panobinostat on key immune response markers, in vitro cell culture results showed panobinostat + IL12 treatment increases MHC I/II on Hepa1‐6 and SB1 cells (Figure 1C). These cytotoxicity and enhanced antigen presentation findings support the utilization of panobinostat and IL‐12 with immunotherapy for synergistic anti‐tumor effects.

### 3.2 PLGA‐PBAE Microparticles can be Efficiently Loaded with a High Dose of Panobinostat and IL‐12 for Co‐Delivery

Following the in vitro finding that panobinostat can potentially sensitize tumor cells for immunotherapy, it was hypothesized that a single dose delivered intratumorally (i.t.) of the chemotherapeutic agent panobinostat and immunomodulatory protein IL‐12 co‐delivered by polymeric MPs can improve antitumor therapeutic efficacy (Graphic Abstract A). Combination therapy MPs are given via i.t. or the intrahepatic artery to mimic TACE in clinical settings. We hypothesized that MPs would accumulate in the tumor mass and release panobinostat in a sustained manner to induce tumor apoptosis. IL‐12, whose tumor retention and cell interaction are enhanced by conjugating IL‐12 to the surface of MPs, promotes immune cell infiltration and inflammatory cytokine secretion. A separate anti‐PD‐1 is dosed to prevent immune evasion from PD‐1 signaling.

**FIGURE 2:**
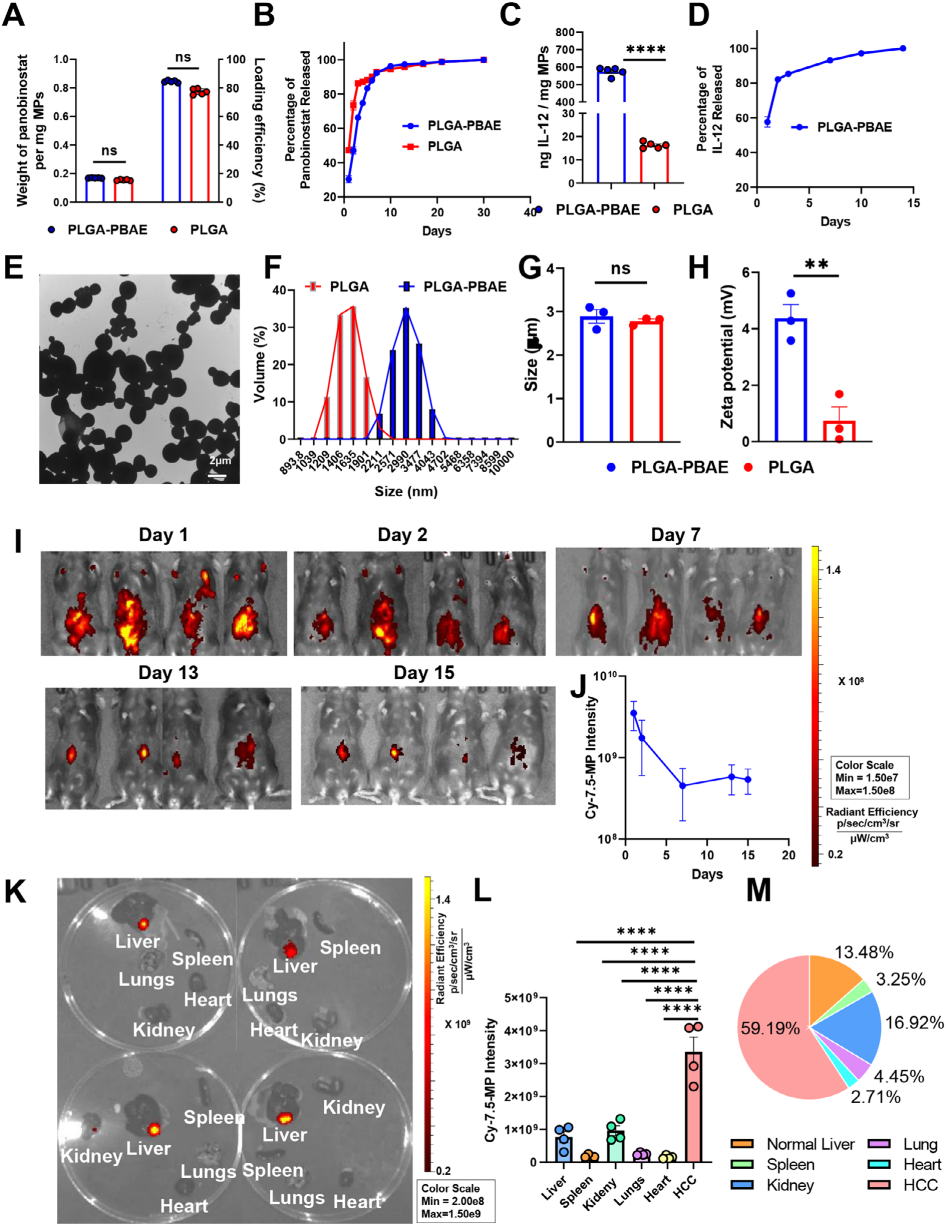
PLGA‐PBAE MPs enable high‐efficiency of loading of panobinostat and conjugation of IL‐12 for co‐delivery, and PPP‐IL12 enables targeted localization and long‐term retention in orthotopic HCC after i.t. injections. (A) Loading efficiency of panobinostat to PLGA‐PBAE vs PLGA MPs (*n*=5). (B) Release profile of panobinostat with sustained release characteristics over the 30‐day tracking period (*n*=5). (C) Mass of IL‐12 conjugated on the PPP MPs (*n*=5). (D) Sustained release and prolonged retention of conjugated IL‐12 from PLGA‐PBAE MPs (*n*=5). (E) TEM of PPP‐IL12 (F,G) Size distribution of PP and PPP MPs (*n*=3). (H) Zeta potential of PP and PPP MPs (*n*=3). (I) IVIS image of Cy7.5‐MP intensity for 15 days after particle injection. (J) Quantification of Cy7.5‐MP intensity of (I). (*n*=4). (K) Cy7.5‐MP distribution in major organs 15 days after particle injection. (L,M) Bar graph and Pie chart of Quantification of Cy7.5‐MP intensity of (K). (*n*=4). Error bars, mean ± SEM. p‐values were calculated using Student's *t*‐tests [(A), (C), (G), and (H)] and ordinary one‐way ANOVA and Dunnett's multiple comparisons test (L). ****p < 0.0001, ****p < 0.0001, ns, not significant.

Polymer blends of PLGA and PBAE showed increased protein conjugation in the design of artificial antigen presenting cells in previous studies [47]. We adapted the similar PLGA/PBAE microparticles to co‐deliver panobinostat and IL‐12 due to their potential to encapsulate panobinostat and conjugate IL‐12 at a high efficiency simultaneously in one particle. We hypothesize that the biodegradable particles are able to act as a sustained‐release depot for panobinostat and enable a sustained in‐situ presence of IL‐12. A hydrophobic PBAE was synthesized via Michael addition reaction of acrylate and amine monomers [36]. MPs were fabricated from PLGA or a blend of PLGA and PBAE by single emulsion, the small molecule panobinostat was loaded into the particles by blending panobinostat with the polymers before emulsification to form panobinostat‐loaded PLGA (PP) MPs or panobinostat‐loaded PLGA/PBAE (PPP) MPs. To co‐deliver panobinostat and IL‐12 with a single‐dose administration, EDC/NHS crosslink chemistry was used to conjugate IL‐12 to PPP or PP MPs to form PPP‐IL12 or PP‐IL12 MPs (Graphic Abstract B). The capacity of the two different MPs to load panobinostat is similar, with ∼20% panobinostat by weight and ∼80% loading efficacy (Figure 2A). The release of panobinostat from both MPs followed a similar sustained release profile for over 1 month (Figure 2B). PPP MPs, which contain the cationic PBAE, showed a 35‐fold increase in the amount of protein that was conjugated on the particles compared with the counterpart PP MPs (Figure 2C). A majority of the conjugated IL‐12 was retained on the surface of the PPP MPs following the first two days of fast release and then slowly released from the particles for over two weeks (Figure 2D). The supernatants of particles from the first two days were retained and analyzed by ELISA to ensure the chemical conjugation of IL‐12 didn't compromise its biological activity (Supplementary Figure S1). Transmission electron microscopy (TEM) showed PPP MPs had a spherical morphology, which is similar to PLGA MPs as previously reported [48] (Figure 2E). The particle sizes and zeta potentials were measured by dynamic light scattering (DLS). Both particles had statistically similar hydrodynamic diameters of 3 µm (Figure 2F,G). While PP MPs possess a neutral surface charge at pH 7.4, PPP MPs maintain a slightly positive surface charge under the same conditions of pH 7.4 (Figure 2H). Overall, both PP MPs and PPP MPs had similar biophysical properties and similar panobinostat loading and release. However, PPP MPs had an improved 35‐fold higher loading of IL‐12 due to the incorporation of PBAE into the MPs, and thus, PPP MPs were selected as the ideal formulation for subsequent use. These data validate that PPP MPs are a competent platform to deliver the small molecule panobinostat and immunomodulatory protein IL‐12 via a single‐dose treatment. To examine the toxicity of our combination therapy of PPP‐IL12 + anti‐PD‐1, a single dose of PPP‐IL12 was administered to a rodent orthotopic HCC model, and anti‐PD‐1 was administered as a separate therapeutic twice a week. The serum was collected 2 weeks after PPP‐IL12 administration. The markers of liver toxicity (such as ALP, AST, ALT, and GGT) and kidney toxicity (such as BUN and creatinine) were within the safety standard (Supplementary Figure S2A–D and Supplementary Table S4). Systemic cytokine analysis was performed on the collected serum for markers of cytokine release syndrome. The PPP‐IL12 + anti‐PD‐1 treated mice didn't show significantly increased cytokine levels of IFN‐γ, IL‐6, CXCL9 (Monokine Induced by Gamma Interferon, MIG), CXCL10 (Interferon gamma‐induced protein 10, IP‐10), CCL2 (Monocyte chemoattractant protein‐1, MCP‐1), or GM‐CSF (Supplementary Figure S2E). And the level of these cytokines were within the normal range. While the serum TNF‐α levels were elevated in the untreated control animals (Supplementary Figure S2E), the abnormal TNF‐α increase was consistent with previous findings on HCC‐bearing animals [49]. The serum TNF‐α levels in the PPP‐IL12 + anti‐PD‐1 treated group were normal.

### 3.3 PPP‐IL12 Enables Long‐Term Orthotopic HCC Retention After i.t. Injections

CY7.5‐labelled PPP MPs were injected i.t. to orthotopic HCC. The fluorescence intensities were measured with IVIS (Figure 2I). The fluorescence signals decreased slowly over 15 days and indicated favorable MP retention in vivo (Figure 2I,J). On day 15 post‐administration, the mice were sacrificed to determine the biodistribution and retention of MPs in major organs. The results showed that up to 60% of MPs were specifically localized in orthotopic HCC, while very few were in normal liver lobes and other major organs with no statistical difference (Figure 2K–M). This indicates an HCC‐specific tumor accumulation and retention over the 15 days. The PPP MPs are a favorable delivery platform as a single‐dose loco‐regional treatment for sustained release of small‐molecule drugs and proteins to HCC.

### 3.4 PPP‐IL12+Anti‐PD‐1 Reduces Tumor Size and Extends Survival in Two Mouse Orthotopic Liver Cancer Models

**FIGURE 3:**
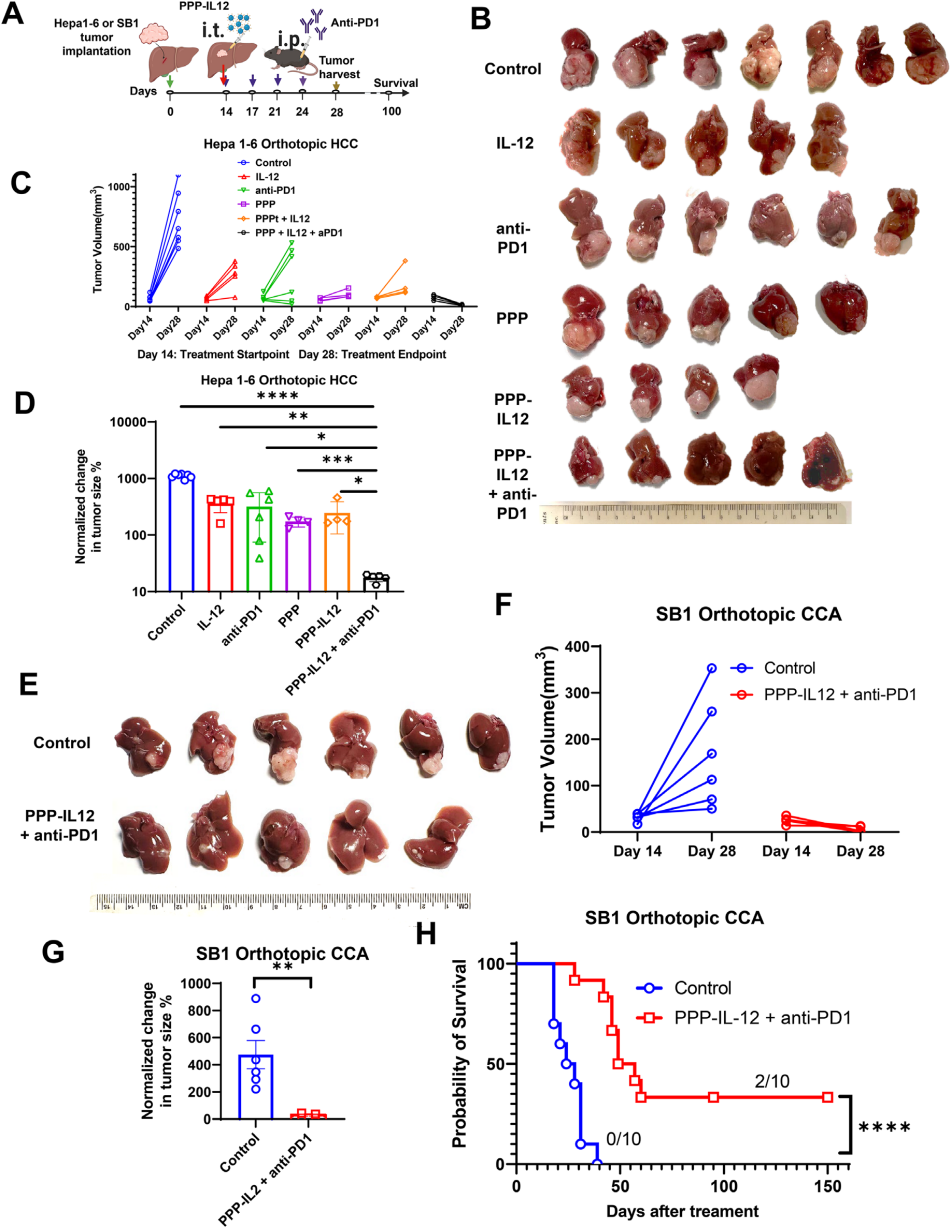
PPP‐IL12+anti‐PD‐1 inhibits tumor growth and extends survival in mouse orthotopic HCC and CCA models. (A) Schematics of the treatment schedule for HCC and CCA orthotopic mouse models. One dose of PPP‐IL12 was given i.t. at day 14, anti‐PD‐1 was given via i.p. twice a week. (B) Gross Images of orthotopic HCC at day 28 (14 days after the single‐dose MPs injection). (C) Tumor volume was measured before and after treatment at day 14 and day 28. (D) End‐point (day 28) tumor size normalized to the start point (day 14) of treatment, respectively. PBS control (*n*=7), IL‐12 conjugated PLGA‐PBAE MPs (*n*=5), anti‐PD‐1 (*n*=6), PPP (*n*=5), PPP‐IL12 (*n*=4) and PPP‐IL12+anti‐PD‐1 (*n*=5). (E) Gross Images of orthotopic CCA at the end point of treatment. (F) Orthotopic CCA tumor volume of PPP‐IL12+anti‐PD‐1 (*n*=5) and PBS control (*n*=6). (G) Normalized CCA tumor size changes. (H) Overall survival curve for mice bearing orthotopic CCA treated with PBS and PPP‐IL12 with anti‐PD‐1 (*n*=10). Mantel‐Haenszel Hazard Ratio is 21.82 with a 95% confidence interval (5.636, 84.49). Error bars, mean ± SEM. P‐values were calculated using a Brown‐Forsythe and Welch ANOVA and two‐stage linear step‐up procedure of Benjamini, Krieger, and Yekutieli multiple comparisons test (D), a Student's *t*‐test (G), or a log‐rank (Mantel–Cox) test (H). *p < 0.05, **p < 0.01, ***p < 0.001, ****p < 0.0001.

To evaluate the therapeutic efficacy of PPP‐IL12 as a loco‐regional strategy of orthotopic liver cancer and compare the treatment with each of the single elements in the combination therapy, orthotopic Hepa1‐6 HCC mice models were subjected to one dose of i.t. MP therapeutics with different cargos (PPP, PPP‐IL12, and IL‐12 conjugated PLGA/PBAE MPs, which include 10 mg panobinostat and 10 µg IL‐12 in MP specifically). The dose of panobinostat in MP is 12 times the free panobinostat oral dose in clinical application [50], the dose of IL‐12 in MPs is at least 10 times of free IL‐12 dose in clinical trials [50, 51]. The same amount of free panobinostat or IL‐12 is lethal to mice without the sustained release system [50, 51]. Anti‐PD‐1 was administered by intraperitoneal injection (i.p.) twice a week (Figure 3A). After 14 days of treatment, the tumors were collected, and the tumor volume was measured (Figure 3B,C). Normalized tumor size was applied to reflect the relative tumor growth over the 14‐day treatment period (Figure 3D). All treatment groups showed tumor growth inhibition compared with the control group, in which the tumor grew over 1,000 times in volume (Figure 3B–D). The combination therapy of PPP‐IL12+anti‐PD‐1 showed the best treatment effect, with an average of 80% reduction in size compared to before treatment. Notably, the single treatment of IL‐12 or anti‐PD‐1 without PPP‐IL12 didn't reduce tumor sizes to the extent of the combination of PPP‐IL12+anti‐PD‐1, which indicates the need of PPP‐IL12 for optimized therapeutic efficacy.

To investigate whether this antitumor efficacy is reproducible in a more immunosuppressive cold liver tumor type, an orthotopic SB1 intrahepatic cholangiocarcinoma (iCCA) mouse model was implemented. Following the same one‐dose regime (Figure 3A), the combination therapy of PPP‐IL12+anti‐PD‐1 reduced the tumor sizes more than 20 times over the 14‐day treatment (Figure 3E–G). To rule out the carrier‐induced effects on the therapeutic efficacy and immune activation, PLGA/PBAE microparticles without panobinostat and IL‐12 (PP) were administered intra‐tumorally to the orthotopic CCA‐bearing mice following the treatment schedule described in Figure 3A. At the endpoint of treatment, the tumor sizes were assessed, and PLGA/PBAE polymeric carriers did not induce any therapeutic efficacy (Supplementary Figure S3A). The tumors and spleens were collected for flow cytometry analysis on local and systemic immune responses. PLGA/PBAE MPs did not have a significant influence on immune cell infiltration to TME (Supplementary Figure S3B–E). We next evaluated the capability of PPP‐IL12+anti‐PD‐1 therapy to prolong the survival of iCCA‐bearing mice. Following the orthotopic CCA inoculation and treatment schedule, the mice received one dose of i.t. PPP‐IL12 and i.p. anti‐PD‐1 twice a week for a total of 40 days, until all the mice in the control group died or were sacrificed according to humane euthanasia criteria. The combination therapy of PPP‐IL12+anti‐PD‐1 prolonged survival by doubling the median survival from 26 days to 53 days, with two of ten mice in the PPP‐IL12+anti‐PD‐1 group classified as long‐term survivors, with tumor‐free survival over 100 days after one dose of PPP‐IL12 and 60 days after the last anti‐PD‐1 administration (Figure 3H). The blood test and pathology studies on the two long‐term survivors showed no significant toxicity in major organs and tumor‐free livers (Supplementary Figure S2 and Supplementary Figure S4). Collectively, these results show that combination therapy of PPP‐IL12 and anti‐PD‐1 resulted in tumor regression in both HCC and CCA orthotopic mouse models and that a single dose of PPP‐IL12 potentially has long‐term antitumor efficacy with extended administration of anti‐PD‐1.

### 3.5 PPP‐IL12 Enables Specific HCC Target Localization and Long‐Term Retention After Intrahepatic Artery Injection in a Rat Orthotopic HCC Model

**FIGURE 4:**
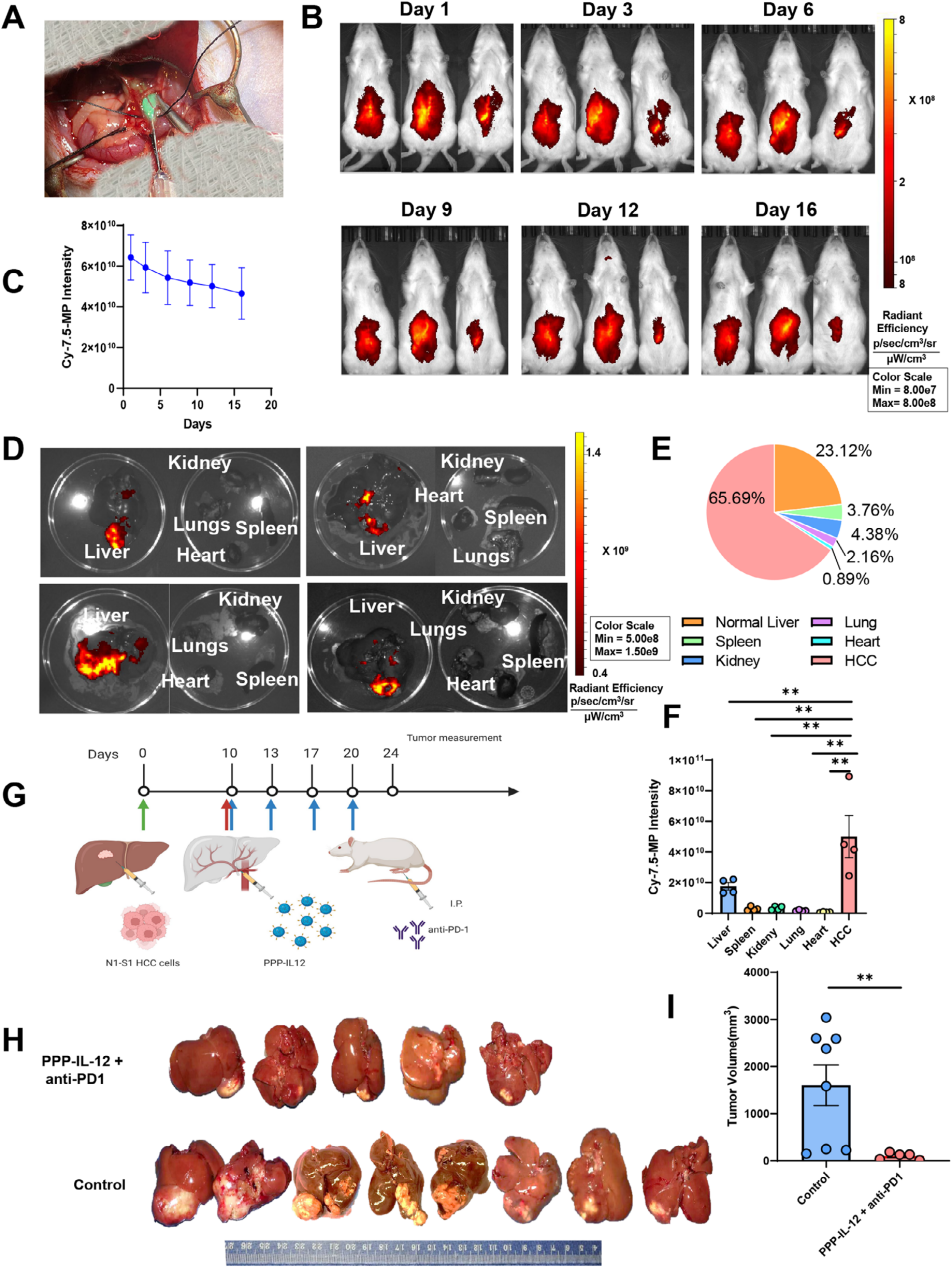
PPP‐IL12 enables targeted delivery and sustained release to orthotopic HCC and reduces tumor sizes in preclinical rat models after intrahepatic artery injections. (A) Image of intrahepatic artery injection of CY7.5‐MPs. (B) IVIS images of Cy7.5‐MPs intrahepatic artery‐injected HCC rats. (C) Quantification of Cy7.5‐MPs in (B) (*n*=4). (D) IVIS images of major organs. (E) Pie charts and (F) bar graphs of quantification of CY7.7‐MPs in HCC site and major organs (*n*=4). Error bars, mean ± SEM. *P* values were analyzed using ordinary one‐way ANOVA and Dunnett's multiple comparisons test. **P < 0.01. (G) Schematics of the treatment schedule in HCC rats. (H) Gross images of livers with orthotopic HCC at the endpoint of treatment (14 days after the single‐dose intrahepatic drug injection). (I) Measured tumor volume. Data were analyzed with a two‐tailed Mann‐Whitney test. **p < 0.01.

To further investigate the in vivo retention and tumor targeting capability of PPP‐IL12 via TACE‐like intrahepatic artery injections, CY7.5‐PPP MPs were delivered via the intrahepatic artery (Figure 4A). Live IVIS Spectrum showed that the Cy7.5‐MPs in the orthotopic HCC regions decreased slowly over 16 days (Figure 4B,C), indicating the in vivo long‐term retention of PPP‐IL12. 16 days after injections, IVIS images of major organs at day 16 post‐injection showed that CY7.5‐MPs signals were primarily in the liver with the orthotopic HCC as the center of signal accumulation (66%), while other organs did not show significant signals (Figure 4D–F). This indicates that PPP‐IL12 specifically accumulated in orthotopic HCC after intrahepatic artery injection. Although the fluorescence signals were diffused throughout the abdominal regions in the whole‐animal imaging, the organ dissections and IVIS images of different organs showed that the fluorescence signals were accumulated in the orthotopic liver cancer regions (Supplementary Figure S5 and Figure 4D–F). The diffused signals are possibly due in part to signal diffusion from the abdominal wall. Collectively, these results demonstrate that PPP‐IL12 acts as a targeted delivery system and enables long‐term retention and sustained release to orthotopic HCC after intrahepatic artery injections.

### 3.6 PPP‐IL12+Anti‐PD‐1 Reduces Tumor Sizes in Orthotopic HCC Rat Models After Intrahepatic Artery Injections

Given the antitumor efficacy of PPP‐IL12 after localized i.t. treatment in two mouse models, we then explored the translational potential of PPP‐IL12 in a more clinically relevant therapy approach similar to TACE in patients. A rat orthotopic HCC model was employed. Ten days after tumor inoculation, a single dose of PPP‐IL12 was administered via intrahepatic artery injection, and anti‐PD‐1 was administered by i.p. twice a week (Figure 4G). The treatment led to dramatically smaller tumor sizes by more than an order of magnitude compared to the control group 14 days after treatment (Figure 4H,I). As a benchmark for the intra‐hepatic arterial injection, a standard clinical TACE treatment agent was administered in orthotopic HCC rat models. The clinical TACE treatment didn't reduce tumor sizes compared to the untreated control (Supplementary Figure S6). Overall, PPP‐IL12 showed robust therapeutic efficacy in preclinical rat models and replicated the findings that we obtained in the two mouse models.

### 3.7 PPP‐IL12+Anti‐PD‐1 Increases Pro‐Inflammatory Cytokine Secretions and Immune Cell Infiltration in Orthotopic HCC and Para‐Cancerous Liver Tissues

**FIGURE 5:**
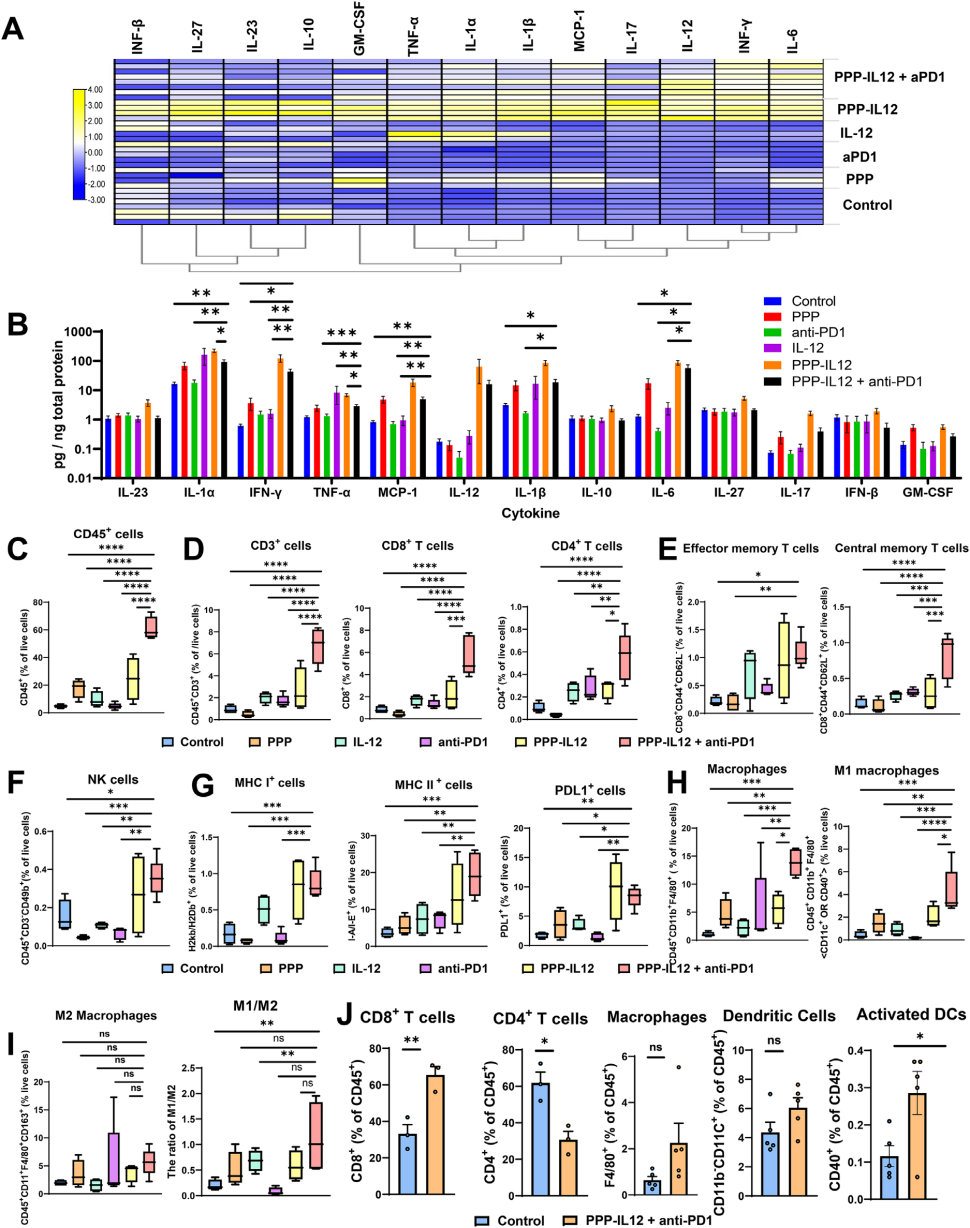
PPP‐IL12+anti‐PD‐1 increases several pro‐inflammatory cytokine secretion and immune cell infiltrations in orthotopic HCC and para‐cancerous liver tissues. (A and B) Cytokine levels in orthotopic HCC tumor lysis are shown in the heatmap (A) and mass normalized to total protein mass (B) of respective groups of untreated control (*n*=7), PPP (n=4), IL‐12 conjugated MPs (*n*=4), anti‐PD‐1 (*n*=5), PPP‐IL12 (*n*=4), and PPP‐IL12+anti‐PD‐1 (*n*=8). (C to I) Flow cytometry analysis on the percentage of lymphocytes (C), CD4+ and CD8+ T cells (D), memory T cells (E), NK cells and NKT cells (F), MHCI/II and PD‐L1 expression (G), and macrophages (H‐I) in para‐cancerous tissues of orthotopic HCC in respective treatment groups of untreated control (*n*=4), PPP treated (*n*=4), IL‐12 conjugated MPs treated (*n*=4), anti‐PD‐1 treated (*n*=5), PPP‐Il12 (*n*=4), and PPP‐IL12+anti‐PD‐1 (*n*=5). (J) Flow cytometry analysis of spleen treated with PPP‐IL12+anti‐PD‐1 vs control. The results indicated CD8+ T cells and activated DCs are upregulated, while CD4+ T cells are downregulated. The total macrophages and dendritic cells showed no difference. A Brown‐Forsythe and Welch ANOVA and Dunnett's multiple comparisons test (B), an ordinary one‐way ANOVA and Dunnett's multiple comparisons tests (C‐I), a Student's *t*‐test (J), *p < 0.05, **p < 0.01, ***p < 0.001, ****p < 0.0001.

To analyze the immune response to the MPs, cytokine levels within the tumor mass from different treatments were measured with a multiplex cytokine detection kit. Pro‐inflammatory cytokines including TNF‐α, IFN‐γ, IL‐1α, IL‐1β, IL‐6 and MCP‐1 were found to be elevated in those that received PPP‐IL12+anti‐PD‐1 therapy, while other cytokines in the measurement didn't show any significant difference (Figure 5A,B).

We postulated that the antitumor efficacy of combination therapy is driven by panobinostat‐induced apoptosis/necrosis, exposing tumor‐associated antigens, as well as by IL‐12 localizing to the TME, boosting immune cell infiltration and proinflammatory immune cell presence. Immune cell infiltration was characterized via flow cytometry on proximal paracancerous tissues (distance to tumor < 1 mm) 7 days after the treatment. While PPP had limited effects on immune cell infiltration, the combination therapy of panobinostat and IL12 within the same MPs facilitated immune infiltration. Paracancerous lymphocytes increased by 6.5‐fold in the combination therapy group compared with the untreated control group, while the PPP group had a lower level of paracancerous lymphocytes (Figure 5C). PPP‐IL12+anti‐PD‐1 increased the proportion of T cells, CD8+ T cells, and CD4+ T cells by 2.4‐, 2.2‐, and 2.5‐fold relative to untreated controls, respectively (Figure 5D). Further, effector and central memory T cells were also increased in paracancerous tissues of combination therapy (Figure 5E). Together, these results indicate an increase in adaptive immune response against HCC. While PPP‐IL12+anti‐PD‐1 treatment elevated adaptive immunity, NK cells were also increased in paracancerous tissues by 1.8‐fold (Figure 5F), indicating an activation of innate immunity against HCC. Given that panobinostat can potentially increase antigen presentation in part due to inducing elevated expression of MHC I/II as observed in vitro, we measured the expression levels of MHC‐I, MHC‐II, and PD‐L1 of paracancerous tissues. The expression of both MHC‐I and MHC‐II was increased following combination therapy (Figure 5G), indicating a potential to enhance immune recognition. PD‐L1 was found to be overexpressed in the PPP‐IL12 treatment groups regardless of anti‐PD‐1 treatment (Figure 5G), and may be a part of the reason for the synergy between PPP‐IL12 and anti‐PD‐1 in orthotopic HCC mouse models. Moreover, macrophages were increased in paracancerous tissues in combination therapy, while the M1 phenotype increased over 10 times from 0.42% to 4.2%, and M2 increased 3 times from 1.9% to 5.6%. M1/M2 ratio, which is an important marker for macrophage polarization, increased from 0.21 to 0.78, indicating M1‐favored polarization (Figure 5H,I). These results suggest increased T cell activation, potentially in part through macrophage antigen presentation. Collectively, these results indicate that PPP‐IL12+anti‐PD‐1 stimulated the secretion of pro‐inflammatory cytokines, increased the infiltration of pro‐inflammatory macrophages, T cells, and NK cells, and facilitated immune recognition by immune cells, activating the immune TME.

In addition, flow cytometry analysis of spleen from PPP‐IL12+anti‐PD‐1 and untreated control showed that the macrophage and dendritic cell population didn't show significant changes, while CD8+T cells and activated DCs increased. (Figure 5J). PPP‐IL12+anti‐PD‐1 potentially influences systemic immunity by increasing the CD8+ T cell population and decreasing the CD4+ T cell population.

### 3.8 PPP‐IL12+Anti‐PD‐1 Induces Intratumoral Immune Cell Infiltration

The increase in proinflammatory cytokines and immune cell infiltration after PPP‐IL12+anti‐PDL treatment suggests immunostimulatory activity in orthotopic HCC regions. To further explore this phenomenon, we employed a CODEX multiplexed fluorescence image to examine the local immune TME with greater granularity, including the specific cell types that infiltrated into the HCC tumors and their spatial relationships [44]. We applied a panel to identify major adaptive and innate immune cell types (Supplementary Table S1) [44, 45].

**FIGURE 6:**
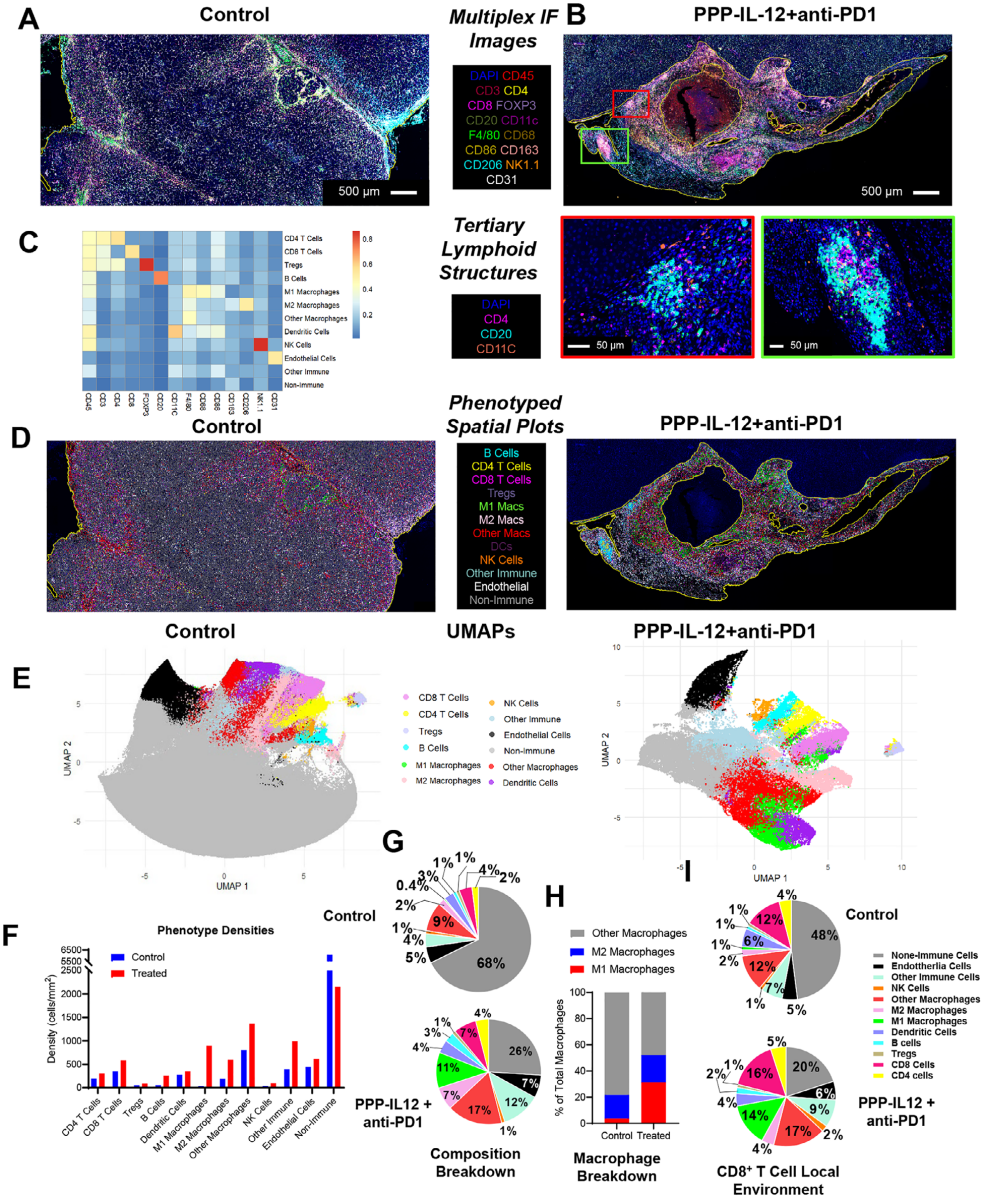
Insights from multiplex proteomics imaging and spatial analysis. (A and B) Multiplex immunofluorescence images of key lineage markers, captured on the CODEX device for untreated control (A) and PPP‐IL12+anti‐PD‐1 treated (B) orthotopic HCC samples (top) and magnified views of the red and green insets showing two well‐defined tertiary lymphoid structures (bottom). Scale bar, 500 µm for HCC sample images or 50 µm for tertiary lymphoid structure images. (C) Heatmap of lineage marker expression profiles for all phenotype populations after cell segmentation in QuPath, min‐max normalization of proteomics values, and cell clustering in the R package FlowSOM. (D) Phenotyped spatial plot showing clusters of immune cells in orthotopic HCC annotations. Scale bar, 500 µm. (E) UMAPs showing major immune subset clusters identified by multiplex proteomics imaging. (F) Densities of all phenotype populations for both untreated control and PPP‐IL12+anti‐PD‐1 treated orthotopic HCC samples. (G) Pie charts describing the compositional breakdown of all phenotype populations in the untreated control (top) and PPP‐IL12+anti‐PD‐1 treated (bottom) HCC annotations. (H) A breakdown of the macrophage subtypes in HCC annotations. (I) Pie charts describing the compositional breakdown of all phenotype populations within a 30 µm radius of CD8+ T cells in both untreated control (top) and PPP‐IL12+anti‐PD‐1 treated (bottom) orthotopic HCC samples.

Multiplex immunofluorescence staining of key lineage markers was applied for untreated control (Figure 6A and Supplementary Figure S7A) and PPP‐IL12 + anti‐PD‐1 treated (Figure 6B and Supplementary Figure S7B) liver HCC. HCC regions were identified by Hematoxylin & Eosin (H&E) staining (Supplementary Figure S8) and annotated in QuPath for further analysis. Necrotic tissue and tissue artifacts were excluded. The R package FlowSOM was used for cell clustering and phenotyping according to lineage marker expression profiles (Figure 6C). The results indicate increased immune cell infiltration in HCC after PPP‐IL12+anti‐PD‐1 treatment, which aligns with flow cytometry data above. The presence of tertiary lymphoid structures (Figure 6B, bottom) after PPP‐IL12 + anti‐PD‐1 therapy indicates an activated immune TME with immune cell infiltration [52], which is also confirmed by H&E staining (Supplementary Figure S8). The diversity of cells in the TME is indicative of higher‐order immune responses occurring and provides support for a robust antitumor immune response. Dimensionality reduction analysis, phenotypic spatial plotting (Figure 6D), and visualization with Uniform Manifold Approximation and Projection (UMAP; Figure 6E) indicate that the antitumor efficacy was perhaps primarily attributable to macrophage clusters, with contributions from CD8 T cell clusters. We further quantified the density of different immune cell populations in both imaged tissues, PPP‐IL12 + anti‐PD‐1 treated HCC TME contained a high diversity of immune cells, including CD8+ T cells, CD4+ T cells, B cells, macrophages, dendritic cells, and a small number of NK cells and Tregs (Figure 6F). We then studied the cell compositions in the imaged tissues. CD8+ T cells and macrophages increased from 4% to 7% and 11% to 35%, respectively, of total cells in the PPP‐IL12+anti‐PD‐1‐treated HCC compared with the untreated HCC (Figure 6G). While all macrophage subtypes increased following PPP‐IL12+anti‐PD‐1 treatment, M1 macrophages particularly increased from 0.4% to 11% of total cells, which was the highest observed increase in cell composition and the major increase in part of the macrophage breakdown analysis. (Figure 6G,H). These spatial findings align with the paracancerous flow cytometry data and the cytokine level measurements, indicating that the PPP‐IL12+anti‐PD‐1 therapy favored M1 macrophage polarization toward a proinflammatory TME. We also analyzed the compositional breakdown of all phenotype populations within a 30 µm radius of CD8+ T cells in both imaged tissues (Figure 6I). The increased composition of M1 macrophages in the CD8+ T cell microenvironment indicates that PPP‐IL12+anti‐PD‐1 increased CD8+ T cell activation through antigen presentation of M1 macrophages. Collectively, these results suggest that the PPP‐IL12+anti‐PD‐1 therapy induced immune cell infiltration into the HCC mass, with antitumor immunity driven in part by M1 macrophage polarization and CD8+ T cell activation.

### 3.9 PPP‐IL12+Anti‐PD‐1 Upregulates the Transcription Level of Cancer Inflammation and Immunity Crosstalk Genes

**FIGURE 7:**
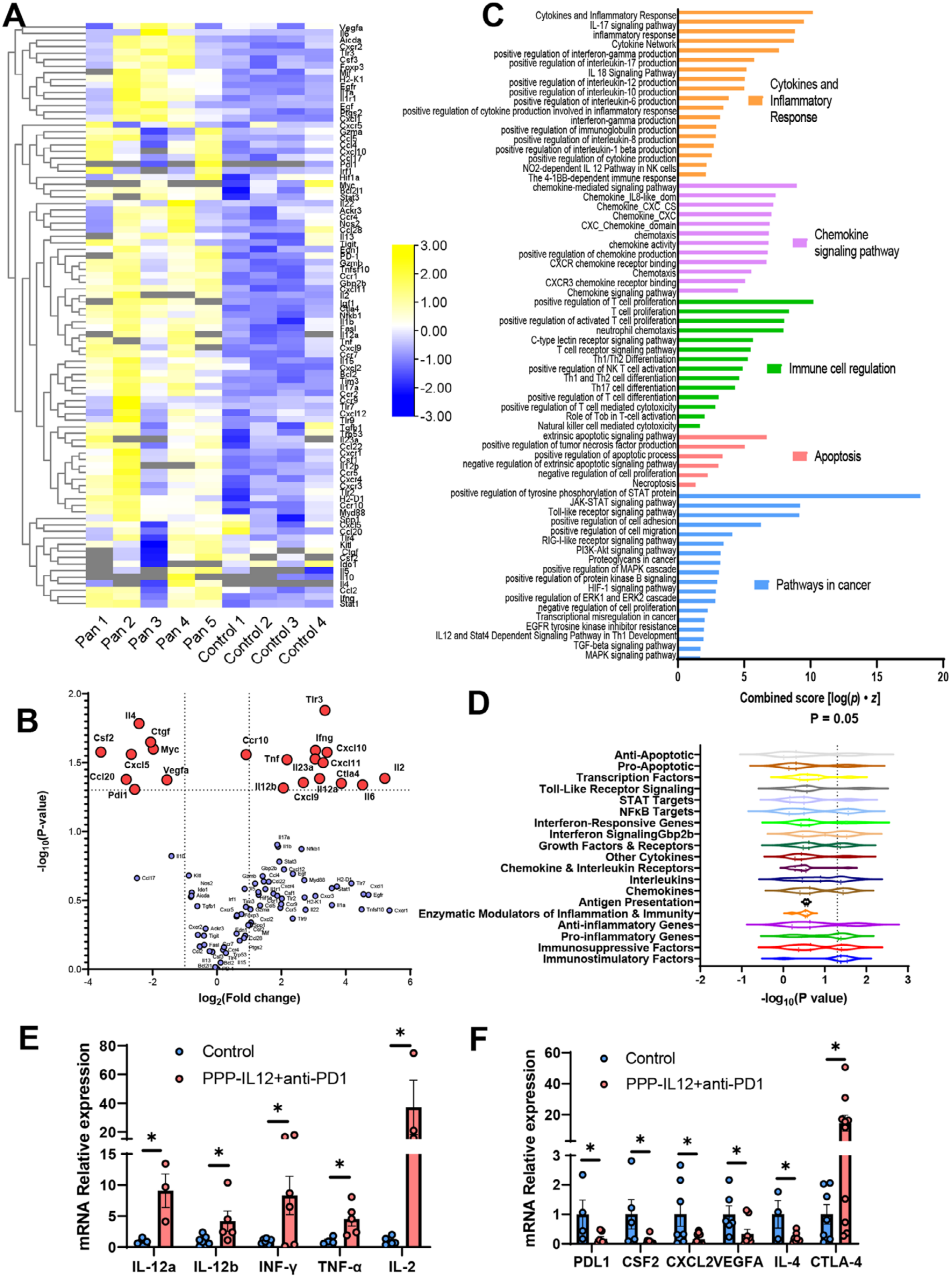
PPP‐IL12+anti‐PD‐1 upregulates the expression of immune and inflammatory response‐related genes and activates related pathways. (A) Heatmap of normalized transcription levels of cytokines and chemokines related to cancer immunotherapies using Mouse Cancer Inflammation & Immunity Crosstalk RT^2^ Profiler PCR Array, (Pan: PPP‐IL12+anti PD‐1, control: Untreated control‐PBS). (B) Volcano plot of *p*‐values of different cytokines and chemokines transcription levels with two‐tailed unpaired *t*‐tests on HCC tumors in untreated control and PPP‐IL12+anti‐PD‐1 treated groups. (C) Pathway analysis by PubMed DAVID functional annotation clustering showing enriched pathways. (D) Transcriptomic analysis identified ontology groups of cancer immune and inflammatory response‐related gene pathways from PPP‐IL12+anti‐PD‐1 vs untreated control. (E) Real‐time RT‐PCR confirmed that several cancer hot immune environment markers are upregulated in PPP‐IL12+anti‐PD‐1‐treated HCC. (F). Real‐time RT‐PCR confirmed that several immune‐suppressive markers are downregulated in PPP‐IL12+anti‐PD‐1‐treated HCC. Notably, CTLA‐4 is upregulated in the PPP‐IL12+anti‐PD‐1 treatment group; the results suggest that combining anti‐CTLA‐4 treatment may be promising for HCC. untreated control (*n*=4–7) and PPP‐IL12 + anti‐PD‐1 (*n*=3–10) groups. Error bars, mean ± SEM. *P* values were calculated using two‐tailed unpaired *t*‐tests (E) and (F).

To determine whether PPP‐IL12+anti‐PD‐1 therapy can regulate the TME, we performed an RT‐PCR array with a panel of mouse cancer inflammation & immunity crosstalk RT^2^ Profiler. Overall, cancer immune and inflammation‐related RNA levels in PPP‐IL12+anti‐PD‐1 treated HCC were higher than those of untreated HCC (Figure 7A). Given the increased transcription levels of immunostimulatory cytokines in HCC of the combination therapy group. Notably, we noticed an increase in mRNA levels of IFN‐γ and TNF‐α, which is consistent with cytokine measurement results (Figure 5B). This observation indicated an immunostimulatory TME. The increased mRNA levels of chemokines, including CXCL9, CXCL11, and CXCL10, further validated the increased infiltration of CD8+ T cells and macrophages into the TME (Figure 7B). Pathway analysis by DAVID functional annotation clustering (davidbioinformatics.nih.gov) on PPP‐IL12+anti‐PD‐1 treated HCCs showed that the IL‐17 signaling pathway and the IFN‐γ pathway dominated cytokine and inflammatory responses. Positive regulation of T cell proliferation and neutrophil chemotaxis dominated immune cell regulation pathways (Figure 7C). When compared with untreated HCCs, transcription levels of molecules related to interferon signaling, interleukins, and chemokines were increased in PPP‐IL12+anti‐PD‐1 treated HCC (Figure 7D), coinciding with the pathway analysis. In addition, key immune markers from PPP‐IL12+anti‐PD‐1 therapy and untreated controls were further measured by RT‐PCR. Immunostimulatory cytokines including IL‐12, IL‐2, IFN‐γ, and TNF‐α, were upregulated (Figure 7E), while immunosuppressive markers of PD‐L1, CSF2, CXCL5, VEGFA, and IL‐4 were downregulated in the combination treatment group (Figure 7F), indicating the immunostimulatory effects on the TME of PPP‐IL12+anti‐PD‐1 therapy. Notably, CTLA‐4 was upregulated in the combination treatment group, indicating the potential target of CTLA‐4 may also further boost the therapeutic efficacy of PPP‐IL12. Together with our previous experiments on TME characterization, these results suggest that PPP‐IL12, combined with anti‐PD‐1, could be a promising therapeutic avenue to reactivate antitumor immunity in HCC.

## 4 Discussion

Cancer immunotherapies have shown great efficacy in various types of cancers by initiating innate and adaptive immune responses to elicit antitumor immunity [53]. Various approaches have been investigated, including aAPCs [32, 36], ICI [53], cancer vaccines [53], and cytokine delivery [33]. However, only a limited number of liver cancer patients have significantly benefited from current cancer immunotherapies, largely due to challenges with the immunosuppressive TME [5, 54]. These hurdles, including the lack of cytotoxic NK and T lymphocyte infiltration into HCC to drive tumor eradication, enrichment of suppressive myeloid populations that inhibit lymphocyte activation, and insufficient antigen presentation due to the lack of tumor‐associated antigens (TAAs) and immune recognition by immune cells, allow tumor cells to escape immune detection [5]. Combination therapies of chemotherapies and immunotherapies have been investigated with the purpose of boosting antitumor efficacy [55]. Here is growing interest in exploring combining immunotherapy with loco‐regional therapies: several studies have shown a potential synergistic effect of combination therapies using either TKIs or VEGF inhibitors with ICI [10, 11]. Immunogenic cell death‐inducing chemotherapeutic agents increase TAA exposure, providing ample targets for the immune system, while the immunotherapies boost presentation of TAAs and activation of antitumor immunity. Cytokine‐based cancer immunotherapy has been promising, and IFN‐α and IL‐2 were approved by the US Food and Drug Administration 20 years ago [56], however, their use is limited due to high toxicity and low efficacy [56]. IL‐12 potently drives antitumor immunity in preclinical studies [22, 27]. However, all clinical trials failed due to severe side effects and limited efficacy [28, 29, 30]. Yet, chemotherapies and immunotherapies have thus far shown limited/no effect as a result of poor tumor targeting, insufficient tumor retention, and systemic toxicity [21, 24]. Polymeric micro‐ and nanoparticles, due to their advanced capabilities of long‐term in vivo retention and sustained release of encapsulated drugs, have been investigated as localized delivery platforms for a range of solid tumors. Loco‐regional treatment for HCC provides intrinsic targeting capabilities for therapeutics to provide local high drug dosage and reduce off‐target toxicity, while biodegradable polymeric particles enable long‐term release of therapeutics into the TME, making particle‐based loco‐regional treatment ideal for a single‐dose targeted combination therapy of HCC.

Here, we designed a multipronged approach by combining an immunostimulatory cytokine and an immunogenic‐cell‐death‐inducing chemotherapeutic drug to target many of the immunosuppressive mechanisms in liver cancers simultaneously. We found that the engineered panobinostat‐IL‐12 MPs had enhanced tumor MP retention, enabled sustained release of panobinostat, enabled prolonged presentation of IL‐12, increased proinflammatory cytokine secretion and signaling in the TME, and increased NK and CD8+ T cell infiltration and activation with the result of tumor regression and prolonged survival in preclinical animal models. Mechanistically, panobinostat enhances tumor apoptosis/necrosis while rendering cancer cells more amenable to immune cell‐mediated killing, in part by upregulating MHC I and antigen presentation. In addition, IL‐12, a cytokine for cancer immunotherapy, aids in immune cell infiltration and activation in the TME.

While there were studies of delivery vehicles for multimodal drug delivery, biodegradable polymeric microparticles have emerged as advanced carrier candidates because of their biocompatibilities, versatile surface for modification, and ease for encapsulation and sustained release of small molecule therapeutics [57]. Lipid nanoparticles are usually considered as delivery platforms for intracellular delivery [58]. Hydrogels have been investigated for intratumoral delivery depots for various therapeutic agents [59], but are generally not considered as carriers for transvascular delivery routes. Polymeric microparticles enable long‐term presence in the TME after localized administration and enable the sustained presence of signal protein IL‐12 in the TME and release of panobinostat as a sustained‐release depot, which makes them an ideal candidate for localized combination therapy. In order to achieve long‐term efficacy with a single‐dose treatment, we fabricated a polymeric MP drug delivery system that enabled tumor retention and sustained release of both panobinostat and IL‐12. Specifically, we first fabricated panobinostat‐loaded MPs by blending PLGA and PBAE. Next, IL‐12 was conjugated to the MPs by EDC‐NHS chemistry to stimulate immune activation. PLGA‐PBAE MPs showed comparable encapsulation capability of panobinostat compared with PLGA MPs, while the conjugation efficiency of 35 times higher IL‐12 on PLGA‐PBAE MPs vs PLGA MPs. PLGA‐PBAE polymeric microparticle blends have been studied for other applications, including for the fabrication of artificial antigen‐presenting cells (aAPCs). In our previous studies, Kelly et al. investigated various formulations of PLGA and PLGA/PBAE microparticle‐based aAPCs [36]. The protein conjugation efficiency was increased from 0.06% to 8.7% as the PBAE content in microparticles increased from 0% to 25%. To maximize the conjugation efficiency and amount of IL‐12 on the PPP‐IL12, polymer blends of 25% PBAE and 75% PLGA were selected as the microparticle delivery vehicles for panobinostat and IL‐12. Similar to the previous results, the selected PLGA/PBAE formulation enabled increased IL‐12 conjugation while encapsulated small molecule panobinostat at a relatively high efficiency of 80%. The increased IL‐12 conjugation of PLGA/PBAE nanoparticles is due to two mechanisms that both occur simultaneously, enhanced physical protein adsorption and improved chemical conjugation efficiency. PLGA/PBAE microparticles adsorb proteins noncovalently in the absence of EDC and NHS chemical conjugation reagents. The enhanced physical adsorption is driven by increased electrostatic charge interactions between regions of positively charged PBAEs and negatively charged proteins. Beyond the increase caused by physical adsorption, there is an additional increase due to increased chemical conjugation not seeing with PLGA‐only particles. This increase is likely due in part to quick hydrolysis of PBAEs at the surface of the particle, and an increased presence of carboxylic acid groups available for protein conjugation [36]. Blending a hydrophobic cationic polymer, PBAE, with anionic PLGA may create a material surface that is especially favorable for protein adsorption at neutral pH [60]. We observed a quicker release in the first days of the in vitro protein release profile. The noncovalently coupled surface proteins can come off from particles faster than the chemically conjugated counterparts. Furthermore, as PLGA and PBAE degrade, the fragments of polymer‐IL‐12 conjugates on the surface come off from the particles, resulting in an initially faster release of IL‐12 at early time points. PPP‐IL12 showed long‐term in vivo retention after administration via i.t. and intrahepatic artery injections. It is important to note that in vitro release experiments are just a model for the in vivo release, and differences in release rates can be due to multiple factors, including the microenvironment (pH gradients, enzymes, etc.), fluid dynamics, and the activities of cells. In our previous studies, we have observed an increased intratumoral accumulation of polymeric particles following the intra‐arterial injection [38, 43]. While the PPP‐IL12 microparticles don't fall in the ideal size range for the enhanced permeability and retention (EPR) effect, it has been reported that high blood flow within the tumor bed naturally funnels a larger proportion of intravascular particles directly into the tumor regions [61]. Moreover, the highly vascularized tumor regions increase the chances of microparticles being mechanically trapped in capillary beds or local tissue spaces [62], considering the lower end of rat capillary sizes is similar to the size of PPP‐IL12. Furthermore, the larger particle size reduces the faster clearance following the injection, enhancing the retention of PPP‐IL12 intratumorally [63]. Therapeutic efficacy of PPP‐IL12 was evaluated in three rodent orthotopic liver cancer models. A single dose of PPP‐IL12, with complementary anti‐PD‐1, enabled tumor regression, promoting durable local antitumor responses in both orthotopic HCC and CCA models. The combination of PPP‐IL12 and anti‐PD‐1 significantly prolonged the survival of mice, resulting in 20% of long‐term tumor‐free survivors in animals. Interestingly, without the prolonged dose of anti‐PD‐1, the tumors became aggressive, and a majority (7 out of 9) of the mice died within 21 days after stopping anti‐PD‐1, while another study found that ICI showed a low response rate [64]. This suggests a potential function of a single dose of PPP‐IL12 in sensitizing the tumor to anti‐PD‐1 for a longer time frame. It is reported that one of the common adverse effects of IL‐12‐based cancer immunotherapy is the induction of systemic cytokines, including IFN‐γ, TNF‐α, IL‐10, CXCL9 (MIG), and CXCL10 (IP‐10) [65, 66]; among the induced cytokines, IFNγ, IP‐10, and TNFα are often the major players of the systemic toxicity [67]. The serum cytokine analysis showed that while the combination therapy treatment group exhibited higher mean serum cytokine levels of IFN‐γ, CXCL9 (MIG), and CXCL10 (IP‐10), the differences were not statistically significant (*p* > 0.05), and the cytokine levels were within normal ranges. Serum TNF‐α levels were higher in untreated HCC‐bearing animals, which is reported as a common effect of HCC [49]. The serum TNF‐α levels were within the normal range in the combination therapy‐treated group.

We found that inflammatory cytokine levels in the HCC increased in animals that received PPP‐IL12, regardless of anti‐PD‐1 treatment, while PPP‐IL12‐anti‐PD‐1 showed improved tumor regression. PD‐L1 in orthotopic HCC was elevated in the PPP‐IL12 treatment group, consistent with previous studies [68]. The overexpression of PD‐L1 indicates the need for anti‐PD‐1 treatment to facilitate combination therapy and strengthen antitumor efficacy. More specifically, IFN‐γ and TNF‐α, antitumor cytokines [69, 70], in the tumor increased in the PPP‐IL12+anti‐PD‐1 treatment group, indicating an activated immune response induced by the combination therapy. HDACi has been investigated to affect macrophage polarization, primarily by inhibiting M2‐type polarization and potentially promoting M1‐type polarization [21]. Flow cytometry results showed that the levels of macrophages in para‐cancerous tissues increased in the PPP‐IL12‐treated group. Based on the size, PPP‐IL12 is susceptible to phagocytosis [71]. It has been reported that PLGA microparticles do not induce changes in phenotype and functionality of dendritic cells upon phagocytosis [72]. Further, unlike tumor cells, phagocytes are usually terminally differentiated cells and relatively insensitive to killing from HDACi panobinostat, which preserves them from chemotherapeutic agents. However, the recruitment of phagocytes, such as macrophages, provides ample opportunities for immune cell infiltration and M1 polarization induced by IL‐12 conjugated on the particles [73]. M1 polarization further induces proinflammatory and antitumor immunity in the TME. The single treatment of IL‐12 or anti‐PD1 without PPP‐IL12 didn't induce proinflammatory cytokine secretion and immune cell infiltration to the extent of the combination of PPP‐IL12 and anti‐PD‐1 did. These results indicate that the immune activation was induced by the synergistic effects of PPP‐IL12 and anti‐PD1. Spatial CODEX analysis further demonstrated that PPP‐IL12/anti‐PD‐1 treatment increased macrophage infiltration in orthotopic HCC and specifically M1 macrophages, indicating a potential polarization effect driven by PPP‐IL12/anti‐PD‐1. The activity of T cells was increased in the PPP‐IL12/anti‐PD‐1‐treated mouse HCC, demonstrated by both flow cytometry analysis and CODEX spatial analysis. We observed an elevation of M1 macrophages and CD8+ T cells in the TME, and the spatial analysis indicates a proximity of M1 macrophages and CD8+ T cells. These results indicate a possible interaction between these cell types, with M1 being antigen‐presenting cells. However, further experiments are in need to confirm the functional relationship between M1 and CD8+ T cells. While we observed several changes in splenocytes, it would be interesting to investigate the abscopal antitumoral effect of the localized treatment in the future. These results were also associated with transcriptional pathway analysis. It is reported that panobinostat regulates the expression of MHC‐I/II on tumor cells [16, 17, 18], and enhances the efficacy of CAR‐T cells against pancreatic cancers [74], with the increased levels of CD8+ T cells facilitating antitumor efficacy. In addition, the presence of tertiary lymphoid structures that we observed on CDOEX imaging indicates a positive response to the immunotherapy [52]. Furthermore, pathway analysis showed an increase in Th17 responses, which has been shown to improve cancer survival in late‐stage HCC after TACE treatment [75]. The toxicity studies showed that the combination therapy didn't induce toxicity to the liver and kidney. Systemic cytokine analysis showed key markers of cytokine release syndrome were within safety ranges. However, future GLP toxicity experiments, including cytokine profiling, hematological evaluation, and dose optimization in additional animal species, are required in future work to further characterize the safety profile and therapeutic effects of the platform.

Our study has several limitations. To evaluate PPP‐IL12 as a TACE therapeutic agent, we performed intrahepatic artery injections in preclinical rat models as a model of TACE‐like therapy to explore the localized drug exposure in HCC rather than to demonstrate artery occlusion in the rodent HCC model following an established rodent model. However, the particles were injected into the common hepatic artery instead of the small hepatic artery branch that selectively feeds the tumor. This limitation, however, suggests that the treatment in patients, where selective catheterization of tumor arteries is possible, would be even more efficacious. While we found that the combination therapy reduced tumor sizes and induced immune cell infiltration and immune activation in the TME, specifically the increase in CD8+ T cells and M1 macrophages, the specific mechanism of tumor killing needs further investigation with T cell‐depleted mouse models and MHC‐deficient mouse models. While the multiple comparisons of different treatment elements of the combination therapy showed the therapeutic efficacy and immunomodulatory function of the combination therapy, the ANOVA‐based comparisons inevitably have compromised robustness due to the limited numbers in each group. Moreover, given that ICI anti‐PD‐1 was necessary to achieve the best therapeutic efficacy in our experiments, it could be helpful to directly conjugate anti‐PD‐1 together with PPP‐IL12 to reduce dosage complexity and systemic toxicity of ICI. Lastly, we found that the transcription level of CTLA‐4 and cytokine levels of IL‐1 and IL‐6 in HCC lysates increased. Thus, a different ICI CTLA‐4 and inhibition of IL‐1 and/or IL‐6 could potentially further improve the antitumor efficacy of PPP‐IL12 combination therapy.

Overall, PPP‐IL12 holds great potential for combination immunotherapy for different types of liver cancers as a single‐dose loco‐regional treatment. Single‐dose PPP‐IL12, combined with anti‐PD‐1, showed robust tumor therapeutic efficacy in multiple preclinical animal models, likely due to the targeted distribution of PPP‐IL12, efficient tumor retention, and sustained release of panobinostat and IL‐12, highlighting the translational potential of the approach. Dose titration studies in larger animals will be needed to establish maximum tolerated particle doses, minimum effective doses before advancing these approaches to patient studies. Collectively, the results suggest that our formulations of polymeric MPs can co‐deliver potent small molecule and protein therapeutics, which can safely overcome immunosuppressive barriers and orchestrate antitumor innate and adaptive immune responses to improve the clinical management of patients with liver cancer.

## Author Contributions

Conceptualization: Hongzhe Yu, Ling Li, Florin M. Selaru, and Jordan J. Green. Methodology: Hongzhe Yu, Ling Li, Florin M. Selaru, and Jordan J. Green. Investigation: Hongzhe Yu, Ling Li, Sachin S. Surwase, Xin Ming M. Zhou, and Hongtao Qian. Visualization: Hongzhe Yu, Ling Li, Sachin S. Surwase, and Xin Ming M. Zhou. Funding Acquisition: Florin M. Selaru and Jordan J. Green. Project Administration: Florin M. Selaru and Jordan J. Green. Supervision: Joel C. Sunshine, Florin M. Selaru, and Jordan J. Green. Writing – Original Draft: Hongzhe Yu and Ling Li. Writing – Review & Editing: Sachin S. Surwase, Xin Ming M. Zhou, Joel C. Sunshine, Florin M. Selaru, and Jordan J. Green.

## Funding

National Institutes of Health (NIH) grants P41EB028239 (JJG), R01CA228133 (JJG), and P41EB024495 (JJG). The Atram Family Family Foundation (FSM). The Johns Hopkins Oncology Tissue Services core, which is also funded by the NIH (P30CA006973).

## Conflicts of Interest

The authors declare no conflicts of interest.

## Data and Materials Availability

All data needed to evaluate the conclusions in the paper are present in the paper and/or the Supplementary Materials.

## Supporting information




**Supporting File**: advs77223‐sup‐0001‐SuppMat.docx.
