# Supplementary material for: Single‐Dose TACE‐Like Injection of Sustained‐Release Panobinostat/IL‐12 Polymeric Microparticles is Effective in Preclinical Liver Cancer Models
Source: Adv Sci (Weinh). 2026 Aug 24:e77223. Online ahead of print. doi: 10.1002/advs.77223 (PMC13502277; doi:10.1002/advs.77223)
Supplement: Supplementary file 1 [file ADVS-9999-e77223-s001.docx]

**Supplementary Figures**

**Supplementary Figure 1. Chemical conjugation does not influence the functional activity of IL-12.** ELISA tests on the supernatants from protein release experiments on day 1 and day 2 and unconjugated IL-12 control concentration. There are not statistical differences between the groups. Error bars, mean ± SEM. Data were analyzed using one-way ANOVA with Turkeys post-test.

**Supplementary Figure 2. Evaluation of systemic hepatic and renal safety profiles following PPP-IL12 + anti-PD-1 combination therapy. (A and B)** Serum biochemistry panels assessing liver and kidney function biomarkers in orthotopic hepatocellular carcinoma (HCC) rodent models following treatment with PPP-IL12 + anti-PD-1 combination therapy. **(C and D)** Serum biochemistry panels assessing liver and kidney function biomarkers in long-term survivors of orthotopic cholangiocarcinoma (CCA) rodent models following the same combination therapy**.** All evaluated parameters remained within normal physiological ranges, as shown **in Supplementary Table 4**. The serum biochemistry analysis was provided by IDEXX BioAnalytics. **(E)** Serum cytokine concentrations were measured to assess systemic inflammatory response and potential toxicity in orthotopic HCC rodent models following treatment with PPP-IL12 + anti-PD-1 combination therapy and untreated control. The results indicate no treatment-induced hepatotoxicity or nephrotoxicity. Error bars indicate mean ± SEM. Statistical comparisons between the control and experimental groups for each cytokine were performed using two-tailed unpaired Student’s t-tests.

**Supplementary Figure 3. PLGA/PBAE MPs (PP) didn’t reduce tumor sizes or induce immune cell infiltration into the TME.** (A) Tumor sizes and gross images of untreated control and PP MPs treated orthotopic CCA cancer. (**B-C**) Flow cytometry analysis on the percentage of T cells **(B),** and macrophages and dendritic cells **(C)** in spleens of orthotopic CCA bearing mice in PLGAS/PBAE (PP) treated and untreated control (n=3). (**D-E**) Flow cytometry analysis on the percentage of T cells **(D)** and macrophages and dendritic cells **(E)** in orthotopic CCA of PLGAS/PBAE (PP) treated and untreated control (n=4). Error bars, mean ± SEM. Data were analyzed with a two-tailed unpaired t-test.

**Supplementary Figure 4. PPP-IL12 + anti-PD-1 treatment enables long-term survival in orthotopic CCA-bearing mice and observes limited systemic toxicity in major organs.** H&E staining showed major organs (lungs, heart, liver, intestine, and kidneys) of the two long-term survivors after PPP-IL12 + anti-PD-1 treatment were normal.

**Supplementary Figure 5. PPP-IL12 accumulation in orthotopic HCC following intrahepatic arterial injection.** IVIS images of rat and major organs 3 days after administration of fluorescently labeled PPP-IL12.

**Supplementary Figure 6. Clinical TACE beads didn’t reduce tumor sizes.** Gross images (left) and tumor sizes (right) of orthotopic HCC following the intrahepatic artery injection of clinical TACE beads. The untreated control tumor volumes are from **Figure 4I**. Error bars, mean ± SEM. Data were analyzed with a two-tailed unpaired t-test.

**Supplementary Figure 7. Multiplex immunofluorescence images of key makers for immune cells via CODEX (PhenoCycler) device. (A)** Untreated control HCC liver lobe **(B)** PPP-IL12+anti-PD-1 treated liver lobe.

**Supplementary Figure 8. Pathology studies show that PPP-IL12+anti-PD-1 induces higher immune infiltration and necrosis of tumor. (A** and **B)** Representative H&E staining showing tumor and infiltrated immune cells in untreated control (A) and PPP-IL12+anti-PD-1 treated (B) mouse orthotopic HCC.

**Supplementary Table 1. Antibodies in CODEX imaging**

| Target | Vendor | Catalog Number | Exposure times (ms) |
| --- | --- | --- | --- |
| CD45 | Cell Signaling Technology | 98819 | 450 |
| CD3 | Cell Signaling Technology | 24581 | 450 |
| CD4 | Abcam | ab271945 | 450 |
| CD8 | Invitrogen | 14-0808-82 | 450 |
| FOXP3 | Cell Signaling Technology | 72338 | 350 |
| CD20 | Cell Signaling Technology | 45839 | 450 |
| CD11c | Cell Signaling Technology | 39143SF | 450 |
| F4/80 | Cell Signaling Technology | 25514 | 150 |
| CD68 | Cell Signaling Technology | 29176 | 450 |
| CD86 | Cell Signaling Technology | 20018 | 150 |
| CD163 | Cell Signaling Technology | 68922BF | 200 |
| CD206 | Cell Signaling Technology | 87887 | 450 |
| NK1.1 | Cell Signaling Technology | 24395SF | 450 |
| CD31 | Cell Signaling Technology | 92841 | 450 |

**Supplementary Table 2. Antibodies in flow cytometry for immune phenotyping**

| Target | Fluorophore | Vendor | Clone | Catalog Number |
| --- | --- | --- | --- | --- |
| CD45 | APC | BioLegend | 30-F11 | 103112 |
| CD45 | FITC | BioLegend | 30-F11 | 103107 |
| CD3 | FITC | eBioscience | 17A2 | 11-0032-82 |
| CD8 | PE/Cyanine7 | BioLegend | QA17A07 | 155018 |
| CD4 | PE | BioLegend | RM4-5 | 100511 |
| CD44 | PE | BioLegend | IM7 | 103023 |
| CD62L | PerCP/Cyanine5.5 | BioLegend | MEL-14 | 104431 |
| CD11b | PE/Cyanine5 | BioLegend | M1/70 | 101209 |
| F4/80 | PE | BioLegend | BM8 | 123109 |
| CD206 | Alexa Fluor 700 | BioLegend | C068C2 | 141734 |
| CD49b | PE | BioLegend | DX5 | 108908 |
| H-2Kb/H-2Db | APC/Fire 750 | BioLegend | 28-8-6 | 114618 |
| CD274 (B7-H1, PD-L1) | PE/Cyanine7 | BioLegend | MIH6 | 153613 |
| I-A/I-E | Alexa Fluor 700 | BioLegend | M5/114.15.2 | 107621 |
| H-2Kb | Alexa Fluor 700 | BioLegend | AF6-88.5 | 116522 |
| CD11c | APC | BioLegend | N418 | 117310 |
| CD40 | PerCP/Cyanine5.5 | BioLegend | 3/23 | 124623 |
| PD-1 | - | Bio X Cell | RMP1-14 | BE0146 |
| Fc | - | BioLegend | 93 | 101320 |

**Supplementary Table 3. Mouse Cancer Inflammation & Immunity Crosstalk RT² Profiler PCR Array primer sequences**

Mus musculus interleukin 2 (Il2)

Forward primer	TGCGGCATGTTCTGGATTTG

Reverse primer	CAAATGTGTTGTCAGAGCCCTTT

Mus musculus interleukin 12b (Il12b)

Forward primer	TGGGAGTACCCTGACTCCTG

Reverse primer	AGGAACGCACCTTTCTGGTT

Mus musculus interleukin 15 (Il15)

Forward primer	CACATCTAACAGCTCAGAGAGAATC

Reverse primer	AGCAAGGACCATGAAGAGGC

Mus musculus colony stimulating factor 2 (granulocyte-macrophage) (Csf2)

Forward primer	ACATGCCTGTCACGTTGAATG

Reverse primer	AAATTGCCCCGTAGACCCTG

Mus musculus cytotoxic T-lymphocyte-associated protein 4 (Ctla4)

Forward primer	AACTGCAGCTGCCTTCTAGG

Reverse primer	GGGTCACCTGTATGGCTTCA

Mus musculus C-X-C motif chemokine ligand 12 (Cxcl12)

Forward primer	AAAGCTTTAAACAAGGGGCG

Reverse primer	GCAGGAAGCGGGGAACTA

Mus musculus C-X-C motif chemokine ligand 5 (Cxcl5)

Forward primer	TGCCCTACGGTGGAAGTCAT

Reverse primer	AGCTTTCTTTTTGTCACTGCCC

Mus musculus indoleamine 2,3-dioxygenase 1 (Ido1)

Forward primer	CCAGTGCAGTAGAGCGTCAA

Reverse primer	TCTGGGTCCACAAAGTCACG

Mus musculus interleukin 10 (Il10)

Forward primer	GCTGTCATCGATTTCTCCCCT

Reverse primer	GACACCTTGGTCTTGGAGCTTAT

Mus musculus interleukin 13 (Il13)

Forward primer	AAGGCCCCCACTACGGTCT

Reverse primer	AGATGCCCAGGGATGGTCTC

Mus musculus interleukin 4 (Il4)

Forward primer	CTCACAGCAACGAAGAACACC

Reverse primer	CTGCAGCTCCATGAGAACACT

Mus musculus interleukin 5 (Il5), mRNA

Forward primer	AATCAAACTGTCCGTGGGGG

Reverse primer	TCCTCGCCACACTTCTCTTTT

Mus musculus macrophage migration inhibitory factor (glycosylation-inhibiting factor) (Mif)

Forward primer	CAGAGGGGTTTCTGTCGGAG

Reverse primer	GTGCACTGCGATGTACTGTG

Mus musculus nitric oxide synthase 2, inducible (Nos2)

Forward primer	TCTAGTGAAGCAAAGCCCAACA

Reverse primer	CCTCACATACTGTGGACGGG

Mus musculus prostaglandin-endoperoxide synthase 2 (Ptgs2)

Forward primer	TGAGTACCGCAAACGCTTCT

Reverse primer	CAGCCATTTCCTTCTCTCCTGT

Mus musculus C-C motif chemokine ligand 2 (Ccl2)

Forward primer	CACTCACCTGCTGCTACTCA

Reverse primer	GCTTGGTGACAAAAACTACAGC

Mus musculus C-C motif chemokine ligand 20 (Ccl20)

Forward primer	TCTGTGTGCGCTGATCCAAA

Reverse primer	TCTTGACTCTTAGGCTGAGGA

Mus musculus interleukin 1 alpha (Il1a)

Forward primer	CCCATGATCTGGAAGAGACCA

Reverse primer	CAAACTTCTGCCTGACGAGC

Mus musculus interleukin 6 (Il6)

Forward primer	GACAAAGCCAGAGTCCTTCAGA

Reverse primer	TGTGACTCCAGCTTATCTCTTGG

Mus musculus interleukin 17A (Il17a)

Forward primer	CTGGACTCTCCACCGCAATG

Reverse primer	GGACCAGGATCTCTTGCTGG

Mus musculus interleukin 22 (Il22), mRNA

Forward primer	GCTCAGCTCCTGTCACATCA

Reverse primer	CAGTTCCCCAATCGCCTTGA

Mus musculus interleukin 23, alpha subunit p19 (Il23a)

Forward primer	ACCAGCGGGACATATGAATCT

Reverse primer	AGACCTTGGCGGATCCTTTG

Mus musculus toll-like receptor 4 (Tlr4)

Forward primer	TCCCTGCATAGAGGTAGTTCC

Reverse primer	TCAAGGGGTTGAAGCTCAGA

Mus musculus activation-induced cytidine deaminase (Aicda)

Forward primer	ACCGATATGGACAGCCTTCTG

Reverse primer	ACCACGTAGCAGAGGTAGGT

Mus musculus histocompatibility 2, D region locus 1 (H2-D1)

Forward primer	TGGTGCTGCAGAGCATTACA

Reverse primer	TGTGCCTTTGGGGAATCTGT

Mus musculus histocompatibility 2, K1, K region (H2-K1)

Forward primer	TGAGAAGGAGAAACACAGGTGG

Reverse primer	GCAGCTGTCTTCACGCTAGA

Mus musculus C-C motif chemokine ligand 4 (Ccl4)

Forward primer	CTGTGCAAACCTAACCCCGA

Reverse primer	AGGGTCAGAGCCCATTGGT

Mus musculus C-C motif chemokine ligand 5 (Ccl5)

Forward primer	GTGCCCACGTCAAGGAGTAT

Reverse primer	TCGAGTGACAAACACGACTG

Mus musculus C-C motif chemokine ligand 22 (Ccl22)

Forward primer	ACCTCTGATGCAGGTCCCTA

Reverse primer	CTTGCGGCAGGATTTTGAGG

Mus musculus C-C motif chemokine ligand 28 (Ccl28)

Forward primer	AGTCATTGCCAGACTCAGTGG

Reverse primer	CCATGGGAAGTATGGCTTCTGA

Mus musculus C-X-C motif chemokine ligand 1 (Cxcl1)

Forward primer	ACTCAAGAATGGTCGCGAGG

Reverse primer	ACTTGGGGACACCTTTTAGCA

Mus musculus C-X-C motif chemokine ligand 2 (Cxcl2)

Forward primer	CCAAAAGATACTGAACAAAGGCA

Reverse primer	CGAGGCACATCAGGTACGA

Mus musculus C-X-C motif chemokine ligand 9 (Cxcl9)

Forward primer	CAGCTCTGCCATGAAGTCCG

Reverse primer	TCCTTATCACTAGGGTTCCTCG

Mus musculus C-X-C motif chemokine ligand 10 (Cxcl10)

Forward primer	CCACGTGTTGAGATCATTGCC

Reverse primer	TCACTCCAGTTAAGGAGCCC

Mus musculus atypical chemokine receptor 3 (Ackr3)

Forward primer	CTCAGCACTGAAGGAGCCTG

Reverse primer	TTCCTGGGAACCACACCTCA

Mus musculus C-C motif chemokine receptor 1 (Ccr1)

Forward primer	ACTCTGGAAACACAGACTCAC

Reverse primer	TCCTTTGCTGAGGAACTGGTC

Mus musculus chemokine (C-C motif) receptor 2 (Ccr2)

Forward primer	AGGAGCCATACCTGTAAATGC

Reverse primer	TGTGGTGAATCCAATGCCCT

Mus musculus chemokine (C-X-C motif) ligand 11 (Cxcl11)

Forward primer	TGGAACATGCAGCCACGTAT

Reverse primer	CCACAGAAGGTAGCGTGGAG

Mus musculus C-C motif chemokine receptor 4 (Ccr4)

Forward primer	CTTTCAGAAGAGCAAGGCAGCTC

Reverse primer TCTGTGACCTCTGTGGCATTC

Mus musculus chemokine (C-C motif) receptor 5 (Ccr5)

Forward primer	TAGATTTGTACAGCTCTCCTAGCC

Reverse primer	ATCCATCCTGCAAGAGCCAGA

Mus musculus C-C motif chemokine receptor 7 (Ccr7)

Forward primer	CATGGACCCAGGTGTGCTT

Reverse primer	CATGAGAGGCAGGAACCAGG

Mus musculus C-C motif chemokine receptor 9 (Ccr9)

Forward primer	TGGAGGCTGGTCTGCATTATC

Reverse primer	CATGCCAGGAATAAGGCTTGTG

Mus musculus C-C motif chemokine receptor 10 (Ccr10)

Forward primer	AAGCCCACAGAGCAGGTCTC

Reverse primer	GGGAGACACTGGGTTGGAAG

Mus musculus C-X-C motif chemokine receptor 1 (Cxcr1)

Forward primer	CCAGCTGGTGCCTCAGATCAA

Reverse primer	GGGCAGCATTCCCGTGAT

Mus musculus C-X-C motif chemokine receptor 3 (Cxcr3)

Forward primer	GCCATGTACCTTGAGGTTAGTGA

Reverse primer	ATCGTAGGGAGAGGTGCTGT

Mus musculus C-X-C motif chemokine receptor 5 (Cxcr5)

Forward primer	AGGCACCAGCACAAACCTTC

Reverse primer	AGGCCAGTTCCTTGTACAGGTC

Mus musculus interleukin 1 receptor, type I (Il1r1)

Forward primer	GGGCACGCCCAGGAGAATA

Reverse primer	TACGTCAATCTCCAGCGACA

Mus musculus kit ligand (Kitl)

Forward primer	GAAGACACAAACTTGGATTATCACT

Reverse primer	TGGAAGATTTGCCACCAGTT

Mus musculus secreted phosphoprotein 1 (Spp1)

Forward primer	CCTGGCTGAATTCTGAGGGAC

Reverse primer	ATCAGTCACTTTCACCGGGAG

Mus musculus tumor necrosis factor (ligand) superfamily, member 10 (Tnfsf10)

Forward primer	CAGCTCATTGAAGAGGTGACTT

Reverse primer	GCTGCCACTTTCTGAGGTCT

Mus musculus colony stimulating factor 1 (macrophage) (Csf1)

Forward primer	GCCCTTCTTCGACATGGCT

Reverse primer	GTTCTGACACCTCCTTGGCA

Mus musculus colony stimulating factor 3 (granulocyte) (Csf3)

Forward primer	CATGAAGCTAATGGCCCTGC

Reverse primer	CTGACAGTGACCAGGGGAAC

Mus musculus epidermal growth factor (Egf)

Forward primer	GGAGGTCCGCTAGAGAAATGT

Reverse primer	TGGGGCATGTGCAGTGATAG

Mus musculus epidermal growth factor receptor (Egfr)

Forward primer	CGCCAACTGTACCTATGGATGTG

Reverse primer	TGCCATTGAACGTACCCAGA

Mus musculus insulin-like growth factor 1 (Igf1), transcript variant 3

Forward primer	TGACCGCACCTGCAATAAAG

Reverse primer	ACTGAAGAGCATCCACCAGC

Mus musculus guanylate binding protein 2b (Gbp2b)

Forward primer	AAGGGCATCTGGATGTGGTG

Reverse primer	TCTGGTTGTCACCCTTCTGC

Mus musculus interferon regulatory factor 1 (Irf1)

Forward primer	TTGACAGCCCTCGAGGAAAC

Reverse primer	CGACGCATGTCAATGCTCAG

Mus musculus myeloid differentiation primary response gene 88 (Myd88)

Forward primer	GATGACCCCCTAGGACAAACG

Reverse primer	ACTCGATATCGTTGGGGCAG

Mus musculus signal transducer and activator of transcription 1 (Stat1)

Forward primer	GATCGCTTGCCCAACTCTTG

Reverse primer	GCAGAGCTGAAACGACCTAGA

Mus musculus toll-like receptor 3 (Tlr3)

Forward primer	TGCGCATATCACAGGCTGAA

Reverse primer	AGGTCCTGCATCCCCTTAGT

Mus musculus BCL2-like 1 (Bcl2l1)

Forward primer	CCTCCTCCCCGACCTATGAT

Reverse primer	TCCAAAGCCAAGATAAGGTTATTCA

Mus musculus myelocytomatosis oncogene (Myc)

Forward primer	GTTGGAAACCCCGCAGACAG

Reverse primer	ATAGGGCTGTACGGAGTCGT

Mus musculus toll-like receptor 2 (Tlr2)

Forward primer	GCATCCGAATTGCATCACCG

Reverse primer	CCTCTGAGATTTGACGCTTTGT

Mus musculus toll-like receptor 7 (Tlr7)

Forward primer	TGGCTCCCTTCTCAGGATGA

Reverse primer	TTGCTGCGAAGAGTGCATTT

Mus musculus toll-like receptor 9 (Tlr9), mRNA

Forward primer	GAGAGACCCTGGTGTGGAAC

Reverse primer	CCTTCGACGGAGAACCATGT

Mus musculus forkhead box P3 (Foxp3)

Forward primer	AAAGCTTAGAGAAGACAGACCCA

Reverse primer	AGTTGCCGGGAGAGCTGAAT

Mus musculus hypoxia inducible factor 1, alpha subunit (Hif1a)

Forward primer	GACAGAGCCGGCGTTTAGG

Reverse primer	CGACGTTCAGAACTCATCCTATTTT

Mus musculus nuclear factor of kappa light polypeptide gene enhancer in B cells 1, p105 (Nfkb1)

Forward primer	CTCTGGCACAGAAGTTGGGT

Reverse primer	TCCCGGAGTTCATCTCATAGT

Mus musculus transformation related protein 53 (Trp53)

Forward primer	ATTCAGGCCCTCATCCTCCT

Reverse primer	CCATGGCAGTCATCCAGTCT

Mus musculus B cell leukemia/lymphoma 2 (Bcl2)

Forward primer	GACTGAGTACCTGAACCGGC

Reverse primer	AGTTCCACAAAGGCATCCCAG

Mus musculus signal transducer and activator of transcription 3 (Stat3)

Forward primer	GACATTCCCAAGGAGGAGGC

Reverse primer	TACGGGGCAGCACTACCT

Mus musculus glyceraldehyde-3-phosphate dehydrogenase (Gapdh)

Forward primer	AACCCTTAAGAGGGATGCTGC

Reverse primer	TCTACGGGACGAGGAAACAC

**Supplementary Table 4. Serum biochemistry test**

| Normal Range | 50-500 U/L | 46-221 U/L | 20-60 U/L | 200–1,500 U/L |  | 0-8 U/L | 200-900 U/L | 0-160 u/L |
| --- | --- | --- | --- | --- | --- | --- | --- | --- |
|  | ALP (U/L) | AST (U/L) | ALT (U/L) | Creatine kinase (U/L) |  | GGT (U/L) | Amylase (U/L) | Lipase (U/L) |
|  | 90 | 104 | 20 | 792 |  | 0 | 564 | 54 |
|  | 70 | 103 | 21 | 679 |  | 0 | 363 | 50 |
|  | 113 | 149 | 36 | 681 |  | 0 | 493 | 51 |
|  | 36 | 90 | 27 | 268 |  | 0 | 398 | 52 |
|  | 64 | 104 | 22 | 559 |  | 0 | 560 | 55 |
|  |  |  |  |  |  |  |  |  |
| Long term survivor 1 | 82 | 52 | 15 | 308 |  | 1 | 553 | 77 |
| Long term survivor 2 | 119 | 58 | 20 | 328 |  | 0 | 588 | 87 |
| Normal Range | 2.2-3.4 g.dL | 0.1 and 0.3 mg/dL | 3.98 to 6.1 g/dL |  |  | 20-40 mg/dL | 0.076 ± 0.002 mg/dL |  |
|  | Albumin (g/dL) | Total Bilirubin (mg/dL) | Total Protein (g/dL) | Bilirubin - Conjugated (mg/dL) |  | BUN (mg/dL) | Creatinine (mg/dL) | Cholesterol (mg/dL) |
|  | 2.5 | 0.1 | 5.9 | 0.0 |  | 24 | 0.04 | 92 |
|  | 2.3 | 0.1 | 5.9 | 0.0 |  | 25 | 0.04 | 76 |
|  | 2.5 | 0.1 | 6.4 | 0.0 |  | 38 | 0.11 | 77 |
|  | 2.2 | 0.2 | 5.4 | 0.0 |  | 21 | 0.00 | 91 |
|  | 2.7 | 0.2 | 5.4 | 0.0 |  | 42 | 0.06 | 101 |
|  |  |  |  |  |  |  |  |  |
| Long term survivor 1 | 3.1 | 0.1 | 5.6 | 0.0 |  | 36 | 0.00 | 94 |
| Long term survivor 2 | 3.0 | 0.1 | 5.9 | 0.0 |  | 31 | 0.10 | 105 |
